# Beneficial Effect of IL-4 and SDF-1 on Myogenic Potential of Mouse and Human Adipose Tissue-Derived Stromal Cells

**DOI:** 10.3390/cells9061479

**Published:** 2020-06-17

**Authors:** Karolina Archacka, Joanna Bem, Edyta Brzoska, Areta M. Czerwinska, Iwona Grabowska, Paulina Kasprzycka, Dzesika Hoinkis, Katarzyna Siennicka, Zygmunt Pojda, Patrycja Bernas, Robert Binkowski, Kinga Jastrzebska, Aleksandra Kupiec, Malgorzata Malesza, Emilia Michalczewska, Marta Soszynska, Katarzyna Ilach, Wladyslawa Streminska, Maria A. Ciemerych

**Affiliations:** 1 Department of Cytology, Institute of Developmental Biology and Biomedical Sciences, Faculty of Biology, University of Warsaw, Ilji Miecznikowa 1, 02-096 Warsaw, Poland; kczaja@biol.uw.edu.pl (K.A.); j.bem@cent.uw.edu.pl (J.B.); edbrzoska@biol.uw.edu.pl (E.B.); areta@biol.uw.edu.pl (A.M.C.); igrabowska@biol.uw.edu.pl (I.G.); p.kasprzycka@biol.uw.edu.pl (P.K.); p.bernas@student.uw.edu.pl (P.B.); r.binkowski@student.uw.edu.pl (R.B.); kinga.jastrzebska@student.uw.edu.pl (K.J.); aleksandra.kupiec@student.uw.edu.pl (A.K.); malgorzata.malesza@student.uw.edu.pl (M.M.); emiliachudek@student.uw.edu.pl (E.M.); m.soszynska4@student.uw.edu.pl (M.S.); kilach@biol.uw.edu.pl (K.I.); krymar@biol.uw.edu.pl (W.S.); 2 Intelliseq Ltd., Stanisława Konarskiego 42/13, 30-046 Krakow, Poland; dzesika.hoinkis@intelliseq.pl; 3 Department of Regenerative Medicine, Maria Sklodowska-Curie National Research Institute of Oncology, W.K. Roentgena 5, 02-781 Warsaw, Poland; katarzyna.siennicka@pib-nio.pl (K.S.); zygmunt.pojda@coi.pl (Z.P.)

**Keywords:** mouse, human, ADSC, differentiation, myogenesis, skeletal muscle, regeneration, IL-4, SDF-1

## Abstract

Under physiological conditions skeletal muscle regeneration depends on the satellite cells. After injury these cells become activated, proliferate, and differentiate into myofibers reconstructing damaged tissue. Under pathological conditions satellite cells are not sufficient to support regeneration. For this reason, other cells are sought to be used in cell therapies, and different factors are tested as a tool to improve the regenerative potential of such cells. Many studies are conducted using animal cells, omitting the necessity to learn about human cells and compare them to animal ones. Here, we analyze and compare the impact of IL-4 and SDF-1, factors chosen by us on the basis of their ability to support myogenic differentiation and cell migration, at mouse and human adipose tissue-derived stromal cells (ADSCs). Importantly, we documented that mouse and human ADSCs differ in certain reactions to IL-4 and SDF-1. In general, the selected factors impacted transcriptome of ADSCs and improved migration and fusion ability of cells in vitro. In vivo, after transplantation into injured muscles, mouse ADSCs more eagerly participated in new myofiber formation than the human ones. However, regardless of the origin, ADSCs alleviated immune response and supported muscle reconstruction, and cytokine treatment enhanced these effects. Thus, we documented that the presence of ADSCs improves skeletal muscle regeneration and this influence could be increased by cell pretreatment with IL-4 and SDF-1.

## 1. Introduction

Skeletal muscles are characterized by the unique ability to regenerate after damages caused by physical or chemical injuries or developing disease. The key role in skeletal muscle repair is played by muscle specific unipotent stem cells, i.e., satellite cells, attached to skeletal myofiber under the basal lamina. In the response to the cues coming from the environment of injured muscle, satellite cells become activated, resume the cell cycle, and differentiate into proliferating myoblasts and finally myocytes, which exit the cell cycle and fuse to create myotubes that mature into new functional myofibers replacing damaged ones (for a review see [1]). Some satellite cells do not undergo differentiation but self-renew to preserve their population. Differentiation of satellite cells to myocytes, myotubes, and finally myofibers is characterized by orchestrated expression of so-called myogenic regulatory factors (MRFs), such as MYOD, MYF5, and MYOGENIN that regulate cell cycle and differentiation, and is associated with the expression of muscle-specific enzymes and structural proteins (for a review see [2]).

Aging or certain pathological conditions, such as myopathies, may lead to malfunction or exhaustion of the satellite cell population, severely affecting muscle regeneration and, consequently, its function. For this reason, various populations of exogenous stem and progenitor cells were and still are tested as a potential source of cells to improve muscle regeneration and function. The first to be evaluated were satellite cells and myoblasts derived from them, but their application in cell therapies appeared to be limited by multiple factors, such as low number of available cells, challenges in sustaining their regenerative potential in vitro, and low survival rate after transplantation (e.g., [3,4,5]). Thus, other types of cells have been targeted by scientists, e.g., mesoangioblasts [6,7,8], pericytes [9,10], muscle-resident interstitial cells [11], muscle side population cells [12], or muscle-derived stem cells (MDSCs) (for a review see [13,14]). However, due to the limitations in their availability, other more readily available cell populations are considered as more promising. Mesenchymal stromal cells (MSCs), which could be isolated from different tissues, are being intensively investigated as a source of cells for regenerative medicine [15,16]. The growing interest in MSCs prompted the International Society for Cellular Therapy (ISCT) to establish minimal criteria to define MSCs, which cover: (i) the expression of CD105 (endoglin), CD73 (ecto-5′-nucleotidase), and CD90 (Thy-1), (ii) the lack of CD11b (Mac-1), CD14, CD31 (PECAM-1), CD34, and CD45 (protein tyrosine phosphatase, receptor type, C also known as PTPRC) antigens, (iii) the ability to adhere to plastic, and (iv) the ability to differentiate into at least adipocytes, osteocytes, and chondrocytes [17,18,19,20]. Interestingly, there is evidence suggesting that regardless of the expression or absence of the abovementioned molecular markers, MSCs isolated from different sources do not possess the same differentiation potential [21]. In the present study we investigated the MSCs isolated from adipose tissue, i.e., adipose tissue-derived stromal cells (ADSCs) in terms of their possible application to support skeletal muscle regeneration. The existence of multipotent ADSCs was reported by Gimble and co-workers [22,23,24,25] and Zuk and co-workers [26,27]. Many studies characterized them as fibroblast-like cells that could be isolated from so-called processed lipoaspirate (PLA). According to the International Federation for Adipose Therapeutics and Science (IFATS), ADSCs should be negative for hematopoietic markers such as CD11b and CD45 and positive for stromal markers such as CD13, CD73, CD90, and CD105 [28]. Some other markers were also mentioned (such as CD36) that allow ADSCs to be distinguished from bone marrow MSCs [29]. ADSCs, such as other MSCs, should differentiate into adipocytes, osteocytes, and chondrocytes. Importantly, human ADSCs maintain differentiation potential and a profile of markers even in long-term cultures [30]. However, it should be noted that obesity, and its related-chronic diseases, or age could negatively impact the proliferation, clonogenicity, and differentiation potential of ADSCs [31,32,33]. Clonal analysis of ADSCs revealed that most ADSC clones (52%) differentiate into two or more lineages, such as adipocytes, osteocytes, chondrocytes, or neuro-like cells [34]. Moreover, the induction of in vitro differentiation of ADSCs into other cell lineages, such as myoblasts and neurons, was also documented [27]. This suggests that ADSCs may undergo multidirectional differentiation, however, the question about their naïve differentiation potential is still open. Studies focusing on the beneficial effect of ADSCs on tissue regeneration suggested that enhanced tissue reconstruction could result not necessarily from ADSC differentiation into specialized tissue cells but due to their paracrine action. Importantly, this ADSC or MSC effect could be modulated by combining cell transplantation with factors which could be beneficial for tissue reconstruction, e.g., for myogenic differentiation or myoblast migration in the case of skeletal muscle. Among such factors are IL-4 and SDF-1. IL-4 activates multiple signaling pathways, such as JAK/STAT or PKB/Akt acting via type I or type II receptors. In skeletal muscles it is type II receptor, i.e., the complex of IL-4Rα and IL-13Rα1, which interacts with IL-4 [35]. It was clearly demonstrated that in the absence of IL-4 or IL-4Rα receptor, formation of skeletal muscles is affected, e.g., myofibers contain fewer nuclei [35]. A similar effect is observed in mice lacking serum response factor (SRF), a transcription factor regulating expression of different genes crucial for proper muscle function such as muscle creatinine kinase or dystrophin, which is also crucial for a proper level of IL-4 expression in skeletal muscles [36]. Treatment of myogenic cells with IL-4 results in elevated expression of β1 and β3 integrins, and, as a result, in enhanced ability for migration both in vitro and in vivo, during skeletal muscle regeneration [37]. However, the complete and exact mechanisms of IL-4 action in myogenic as well as other cells are not fully known. SDF-1 (stromal cell derived factor-1), encoded by *Cxcl12*, also known as chemokine CXCL12, i.e., the factor which is ubiquitously expressed in embryonic and adult tissues [38,39], binds to receptors belonging to the CXCR family, i.e., CXCR4 and CXCR7 [40,41]. Signaling mediated by CXCR4 plays an important role in stem and progenitor cell migration during embryonic development and also in adult tissues [42]. CXCR7 receptor (also known as atypical chemokine receptor 3, ACKR3), except SDF-1, also binds ITAC and MIF [41]. CXCRs belong to the family of seven transmembrane domain G-protein-coupled receptors (GPCRs) which activate multiple signaling pathways, including the ERK1/2 or PI3K-AKT-mTOR and JAK-STAT pathways. CXCRs were shown to form homo- or hetero-oligomers in a ligand-inducible manner, resulting in a complex crosstalk between many signaling pathways. CXCR4 and CXCR7 form complexes constitutively recruiting β-arrestin and impairing G-protein-mediated signaling. In CXCR4/CXCR7 heterodimers, CXCR7 acts as β-arrestin signaling enhancer [43].

Previously, we showed that skeletal muscle reconstruction could be improved by SDF-1 as the factor stimulating cell migration and homing, injected directly to the injured muscle or by transplantation of MSCs pretreated with this factor [44,45,46,47]. We also showed that IL-4, which influences myogenesis by stimulating myogenic cell migration and fusion [35,37], can increase the ability of other cells, specifically ESCs, to undergo myogenic differentiation by increasing the expression of early myogenic genes (*Msgn1, Pax3, Pax7*) [48]. Taking into consideration available data, we hypothesized that IL-4 and SDF-1 treatment, alone or combined, will have a beneficial effect on ADSC features, improving their migration and regeneration-supporting potential. Next, we aimed to test the similarities and differences between ADSCs originating from mouse and human.

## 2. Materials and Methods

Human adipose tissue was purchased from Lonza (PT-50-06) and Rooster (MSC-021, MSC-022), or supplied by the Horizon Medical Center after routinely performed esthetical liposuctions. Adipose tissue samples were collected from the „waste material” before its disposal, the procedure did not influence the clinical indications for liposuction, nor patient’s treatment, according to the approvals of Ministry of Health MZ-PZ-TSZ-025-12792-9/MN/13 and PZT.04061.17.2018MK. All donors gave their informed consent for inclusion and use of leftover material for research purposes (consent HMC/ZGP/2016/12/01, HMC/ZGP/2017/09/01, HMC/ZGP/2017/11/01). All procedures involving mice were approved by the Local Ethics Committee No. 1 in Warsaw, Poland—permission numbers: 626/2014 and 493/2017.

### 2.1. Cell Isolation and Culture

Mouse adipose tissue was isolated from C57Bl/6N male mice (age 6-8 weeks), transferred to betadine solution (8 µl/mL, EGIS Poland sp. z o.o.) in phosphate buffer saline (PBS), and then washed with PBS. Mouse adipose tissue-derived stromal cells (mADSCs) were isolated by digestion of fragmented adipose tissue with 0.2% type I collagenase solution (Sigma-Aldrich) for 90 min at 37 °C. Cells were cultured in high glucose Dulbecco’s modified Eagle’s medium (DMEM, Invitrogen) supplemented with 10% fetal bovine serum (FBS, Invitrogen) and 10 μg/mL gentamycin (Sigma-Aldrich) in 5% CO_2_ at 37 °C. Culture medium was replaced every 2–3 days, and cells were passaged after reaching 70% confluency. For further experiments, cells from passages 3-6 were used. Human adipose tissue-derived stromal cells (hADSCs) were isolated as described previously [49]. Briefly, lipoaspirate diluted in PBS (Life Technologies) was incubated with 0.025% collagenase (Sigma-Aldrich) for 1.5 h in 37 °C, then washed with PBS containing 2% human albumin (Kendrion), and centrifuged (400× *g*, 10 min). The resulting cell pellet was suspended in high glucose DMEM supplemented with 10% FBS.

### 2.2. Cell Treatment with IL-4, SDF-1, and IL-4 and SDF-1

Mouse or human ADSCs were cultured at density of 5 × 10^3^/cm^2^ in high glucose DMEM supplemented with 10% FBS and 10 μg/mL gentamycin in the absence or presence of either 10 ng/mL of species-specific IL-4 (recombinant mouse IL-4 protein I1020, Sigma-Aldrich; recombinant human IL-4 protein ab83686, Abcam) or 25 ng/mL of species-specific SDF-1 (recombinant mouse SDF1α protein ab51939, Abcam; recombinant human SDF-1α protein ab73461, Abcam) or both of them. Culture medium supplemented with IL-4 or/and SDF-1 was replaced every 24 h to keep the required concentration of the abovementioned factors. The morphology of cells was analyzed using a Nikon Eclipse, TE200 microscope with Hoffman contrast and NIS Elements software. Cell proliferation was assessed for control and treated mADSCs every day from day 3 to 7 of the culture and for control and treated hADSCs every day from day 1 to 7 of the culture. For qPCR and immunocytochemistry analysis control and IL-4 treated and SDF-1 treated mADSCs were collected after 24 h, 72 h, and 7 days of the culture. Human ADSCs treated with either IL-4 or SDF-1 or both factors as well as control cells were collected after 48 h of culture for qPCR and immunocytochemistry, as well as for ELISA and mRNA sequencing analysis. 

### 2.3. Migration Assay

Migration of ADSCs was analyzed using scratch wound healing assay [50]. Briefly, mouse or human ADSCs were cultured in high-glucose DMEM supplemented with 10% FBS and 10 μg/mL gentamycin in the absence or presence of either 10 ng/mL of IL-4 or 25 ng/mL of SDF-1 until 90% of confluency. Next, the cells were scratched from the plate using a plastic automatic pipette tip to create the “wound” (0 h). The wound healing manifested by the ability of cells to refill the created gap was monitored after 6 and 24 h of culture. At 0, 6, and 24 h the culture was photographed and the area which lacked cells (non-invaded area) was measured and presented as the proportion of the whole photographed area. Three independent experiments were performed.

### 2.4. ELISA

For ELISA analysis culture supernatants were collected after 48 h of the culture of either control, IL-4 or SDF-1, or IL-4 and SDF-1 treated human ADSCs and analyzed with SunRed Biotechnology Company ELISA kits, according to the manufacturer’s instruction. The following antigens were analyzed: MCP1/CCL2; IL-11; BDNF; BRAK/CXCL14; IGFBP5; TGFβ; GCP2/CXCL6; Eotaxin 3/CCL26; MIP2/CXCL2; Eotaxin/CCL11. Culture medium, i.e., high glucose DMEM with 10% FBS and 10 μg/mL gentamycin was also included and described in the article as M (medium). The plates were analyzed with Gen5 Microplate Reader (BioTek). 

### 2.5. Co-Culture of ADSCs with C2C12 Myoblasts

C2C12 myoblasts (European Collection of Cell Cultures ECACC no. 91031101, passage no. 13) were cultured in medium supporting their proliferation, i.e., high glucose DMEM supplemented with 10% FBS and antibiotics. For co-culture experiments, 25 × 10^3^ of mouse C2C12 myoblasts and 25 × 10^3^ of mADSCs were seeded on gelatin-coated sterile coverslips and cultured in high glucose DMEM supplemented with 10% of FBS and 10 μg/mL gentamycin, in the presence or absence of either IL-4 or SDF-1 for 7 days. The medium was replaced every day to maintain a required concentration of cytokines. Mouse ADSCs used for the co-culture were earlier labelled either with Orange CMRA (Life Technologies) or BacMam GFP Transduction Control (Invitrogen) according to the manufacturer’s instructions. In another set of experiments, 25 × 10^3^ of hADSCs were cultured for 2 days in the presence or absence of either IL-4, SDF-1 or both factors (with medium replacement after 24 h), followed by co-culture with C2C12 myoblasts in high glucose DMEM with 10% of FBS and 10 μg/mL gentamycin until myotubes were visible, i.e., 7–14 days. Cells were fixed with 3% paraformaldehyde (PFA, Sigma-Aldrich) and proceeded for immunocytochemistry analysis. Orange CMRA and BacMam GFP allowed for the identification of hybrid myotubes, i.e., those generated by mouse ADSCs fusing with C2C12 myoblasts. In the co-culture of hADSCs with C2C12 myoblasts, hybrid myotubes were identified by immunostaining of human nuclear antigen (mouse monoclonal antibody ab191181 Abcam) followed by an appropriate secondary antibody. Nuclei were stained with Hoechst 33342 (Sigma-Aldrich) or DRAQ5 (Biostatus Limited) diluted 1:1000 in PBS. To visualize the localization of myotubes, co-cultures were stained with either antibodies against skeletal muscle myosin (M7523, Sigma-Aldrich) or anti-skeletal muscle marker (12/101-c, DSHB). For each experimental group the number of hybrid myotubes was counted from at least five fields of view. Data are the mean ± standard deviation of three biological replicates.

### 2.6. Preparation of Cells for Transplantation

Mouse or human ADSCs were cultured in high glucose DMEM supplemented with 10% FBS and 10 μg/mL gentamycin in the absence or presence of either 10 ng/mL of IL-4 or 25 ng/mL of SDF-1 or both factors for 48 h. The medium was replaced after 24 h, and in case of mADSCs, it was then supplemented with BacMam GFP Transduction Control (Invitrogen) and left overnight, in accordance with the manufacturer’s instruction. Next, cells were washed with PBS and detached with 0.05% trypsin (Invitrogen). Cells were collected, centrifuged, and suspended in 0.9% NaCl solution at 5 × 10^7^ cells/mL density for transplantation studies. 

### 2.7. Skeletal Muscle Injury

After anesthesia of the mouse (12-week-old male C57BL/6N or NOD/SCID) with isoflurane gastrocnemius, muscles were damaged by the injection of 50 μL of 10 μM cardiotoxin (Latoxan). After 24 h, injured muscles were injected with 20 μL of 0.9% NaCl solution containing either 1 × 10^6^ of control (i.e., untreated) or treated ADSCs as described above. Mouse ADSCs labelled with BacMam GFP were transplanted to injured muscle of C57BL/6N males, and human ADSCs were transplanted to injured muscles of NOD/SCID mice. In control experiments injured muscles were injected with 20 μL of 0.9% NaCl solution only or muscles were not treated, i.e., remained intact (hereafter referred to as NIM, i.e., non-injured/injected muscle). Each variant of the experiment was performed at least in three biological repeats. After the procedure, animals were kept under standard conditions with free access to food and water. 

### 2.8. Histological Analyzes—Myofiber Number and Connective Tissue Area

Mice were sacrificed using spinal cord dislocation. Muscles and animals from which the muscles were taken were weighed. The isolated muscles were frozen in isopentane, cooled in liquid nitrogen, and stored at −80 °C. The frozen mouse muscles were cut into sections of 10 μm thickness using cryostat (Microm HM505N). Cross-sections were placed on slides and after drying were stored at 4 °C. Sections were hydrated in PBS (10 min) and then stained either with hematoxylin and eosin (Sigma-Aldrich) or Masson’s Trichrome (Sigma-Aldrich), according to the manufacturer’s instructions. The stained sections from each muscle were analyzed using a Nikon TE200 microscope (Zeiss) and NIS Elements software. Next, the mean area of 100 regenerating myofibers as well as the area occupied by the connective tissue in relation to the area of the entire section were determined using ImageJ software for each muscle. At least three independent biological samples for each variant were analyzed. The obtained data were averaged and presented on a graph.

### 2.9. Localization of Transplanted Cells 

Ten micrometer-thick sections were obtained as described above. Cross-sections of control or mouse muscles injected with mADSCs or hADSCs isolated after 7 (CTX7), 14 (CTX14), and 30 (CTX30) days of regeneration were hydrated in PBS (10 min), fixed with 4% PFA (15 min), and washed again in PBS (10 min). Next, sections were subsequently incubated in 0.2% Triton-X100 (Sigma-Aldrich) in PBS (5 min), 0.25% glycine (Sigma-Aldrich) in PBS (30 min), and finally in 3% bovine albumin solution (BSA) and 5% FBS in PBS (1 h). Next, sections obtained from mouse muscles injected with mADSCs were incubated in anti-GFP antibody (ab6556 Abcam) diluted 1:500 in 3% BSA in PBS (2 h). After washing in PBS, sections were incubated in 0.3% hydrogen peroxidase solution in PBS (15 min) and then in anti-rabbit IgG-HRP antibody, diluted 1:400 in 3% BSA in PBS (1 h). Finally, sections were washed with PBS and incubated in ImmPACT DAB Chromogen solution (Vector Laboratories) until assumed a brown stain appearance, then washed in water, stained with hematoxylin QS (Vector Laboratories) for 45 sec, dehydrated, and mounted with VectaMount (Vector Laboratories). Obtained specimens for each muscle were analyzed using a Nikon TE200 microscope and NIS Elements software. In the case of sections obtained from mouse muscles injected with hADSCs incubation in 3% BSA and 5% FBS in PBS, was followed by staining with anti-human nuclear antigen (mouse monoclonal antibody ab191181 Abcam) diluted 1:100 in 3% BSA in PBS (2 h) and then appropriate secondary antibody and DRAQ staining. Specimens were mounted with fluorescent mounting medium (Dako Cytomation) and analyzed using an Axio Observer Z1 LSM 700 scanning confocal microscope (Zeiss) equipped with ZEN software (Zeiss). 

### 2.10. qPCR

RNA was isolated from ADSCs using a High Pure RNA Isolation Kit (Roche) and from muscles using an mirVana miRNA Isolation Kit (Thermo Fisher Scientific), according to the manufacturer’s instructions. Reverse transcription was performed using 0.5 μg total RNA and a RevertAid First Strand cDNA Synthesis Kit (Thermo Fisher Scientific), according to the manufacturer’s instruction. qPCR was performed using specific TaqMan^®^ probes (listed in Table S1), TaqMan Gene Expression Master Mix (Thermo Fisher Scientific), and a Light Cycler 96 instrument (Roche). Data was collected and analyzed with Light Cycler 96 SW1.1 software (Roche). Analysis of relative gene expression was performed according to Livak [51]. 

### 2.11. Immunolocalization 

Control and treated mouse and human ADSCs were fixed with 4% PFA (15 min) after 1, 3, and 7 days of the culture. Cells were washed in PBS (10 min), incubated in 0.2% Triton-X100 in PBS (5 min), 0.25% glycine in PBS (30 min), 3% BSA in PBS (1 h), and finally in selected primary antibodies solution in 3% BSA in PBS (either at room temperature for two hours or overnight at 4 °C). The following primary antibodies were used: anti-CD105 (mouse monoclonal ab11414, Abcam); anti-CD90 (mouse monoclonal ab225, Abcam); anti-CXCR4 (rabbit monoclonal ab124824, Abcam); anti-CXCR7 (rabbit polyclonal ab117836, Abcam); anti-IL4R (rabbit polyclonal NBP1-00884, Novus Biologicals); anti-IL13R (rabbit polyclonal NBP1-61690, Novus Biologicals); anti-MYOD (rabbit polyclonal c-20, Santa Cruz Biotechnology), and anti-CD9 (rabbit monoclonal ab92726, Abcam). After washing in PBS, cells were incubated with appropriate secondary antibodies, diluted 1:200 in 3% BSA in PBS (2 h) and then in Hoechst 33342 or DRAQ5 diluted 1:1000 in PBS (5 min). Muscle cross-sections were rehydrated for 10 min in PBS and then fixed for 10 min in 3% PFA in PBS. Next, sections were permeabilized with 0.1% Triton X-100 in PBS (Sigma-Aldrich) and incubated with 0.25% glycine for 15 min (Sigma-Aldrich). Non-specific binding of antibodies was blocked with 3% bovine serum albumin (BSA, Sigma-Aldrich) in PBS, at room temperature, for 30 min. Next, sections were incubated with selected primary antibodies diluted 1:100 in 3% BSA in PBS, overnight. The following primary antibodies were used: anti-CD45 (rat monoclonal ab25386, Abcam), anti-CD68 (rat monoclonal ab53444, Abcam), anti-CD163 (rabbit monoclonal ab182422, Abcam), and anti-GFP (rabbit polyclonal ab6556, Abcam). After PBS washing, sections were incubated at room temperature with appropriate secondary antibodies diluted 1:200 in 3% BSA in PBS for 2 h. Cell nuclei were visualized using incubation with Hoechst 33342 or DRAQ5 diluted 1:1000 in PBS for 10 min. All specimens were mounted with fluorescent mounting medium (Dako Cytomation) and analyzed using an Axio Observer Z1 LSM 700 scanning confocal microscope equipped with ZEN software.

### 2.12. Next Generation RNA Sequencing (NGS)

RNA sequencing was performed for four hADSC lines in four experimental groups: control, IL-4-treated, SDF-1-treated, and IL-4/SDF-1-treated ADSCs. RNA was extracted from 1 × 10^6^ cells per each sample using an RNeasy Mini Kit (Qiagen) and a QIAshredder homogenizer (Qiagen), according to the manufacturer’s protocol. RNA quality and concentration were assessed in a NanoDrop microvolume spectrophotometer (ThermoFisher Scientific) and Bioanalyzer 2100 (Agilent). Only samples with RNA integrity number, i.e., RIN values ≥ 8 qualified for further library preparation. RNA samples were diluted in nuclease free water and were subjected to reverse transcription using ProtoScript II Reverse Transcriptase (NEB). NGS sequencing was outsourced to OpenExome company. The quality of the NGS data was verified using FastQC (v. 0.11.5). The RNA-seq reads were aligned to GRCh37.p13 using Hisat2 (v. 2.0.5). The transcript and gene FPKM (fragments per kilobase of transcript per million fragments mapped) levels were quantified using Cuffquant, Cuffnorm (v. 2.2.1), and GTF from the Ensembl (v. 75) gene database. Statistical significance was analyzed using one-way analysis of variance (ANOVA) on log2(1 + x) values. The false discovery rate (FDR) was estimated using the Benjamini–Hochberg method. All statistical analyses were performed using R software (v. 3.4.3). Resulting data were deposited in the NCBI Sequence Read Archive (SRA) with accession number PRJNA623071. 

### 2.13. Statistical Analysis

Results were analyzed using GraphPad Prism 8.0.2 software (San Diego, CA, USA). First, we performed a Shapiro-Wilk normality test. Depending on the results we either used a parametric non-paired two-way ANOVA test or a Student’s t-test (data with normal distribution) or a nonparametric non-paired Kruskal-Wallis test (data with non-normal distribution). A one-way ANOVA test was performed for the results of RNA sequencing. The test performed is described in each figure legend. The differences were considered statistically significant when *p* < 0.05. Data are shown as mean ± standard deviation.

## 3. Results

The aim of our study was to test the hypothesis whether IL-4 or/and SDF-1 could enhance the potential of adipose tissue-derived stromal cells (ADSCs) originating from mouse (mADSCs) and human (hADSCs) to undergo myogenic differentiation and/or improve skeletal muscle regeneration. To do so we performed molecular and cellular analyses of mouse and human ADSCs cultured in vitro as well as analyses of skeletal muscles into which such cells were transplanted. In each case, we compared control ADSCs and those that were subjected to cytokine treatment. In in vitro studies we analyzed cells cultured up to 14 days, and in the case of in vivo studies, our analyses covered 30 days of skeletal muscle regeneration. 

### 3.1. Mouse ADSC Response to IL-4 or SDF-1 Treatment In Vitro

**Figure 1:**
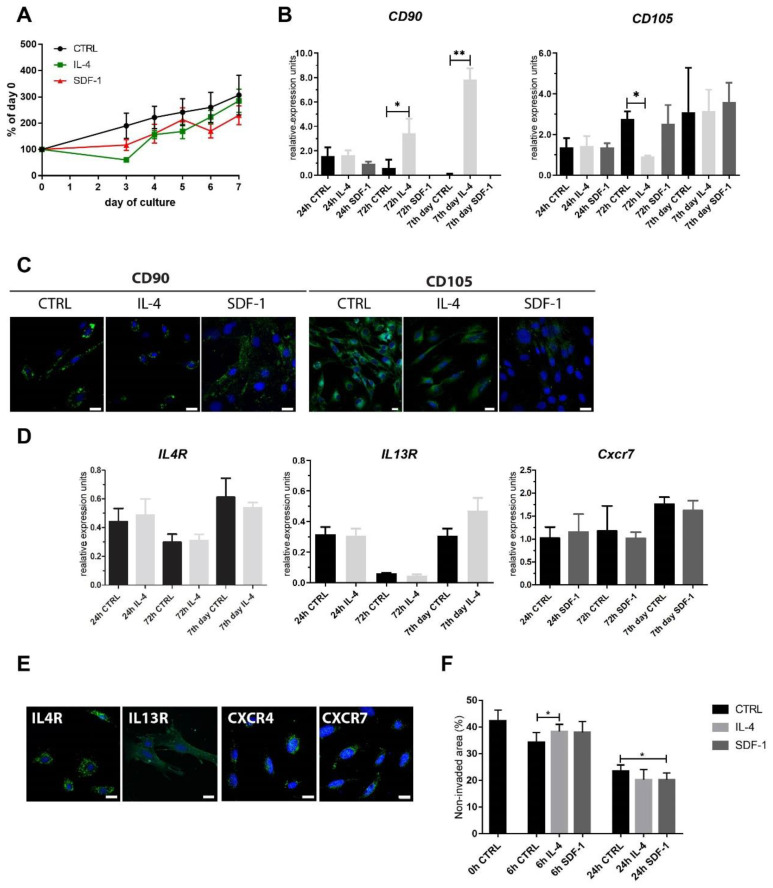
Characterization of mouse adipose tissue-derived stromal cells (mADSCs) cultured under control conditions or in the presence of IL-4 or SDF-1. (**A**) Growth curves of mADSCs cultured for 7 days; data shown as a proportion of the number observed at day 0. (**B**) Analysis of the level of mRNAs encoding CD90 and CD105. Expression was related to the levels observed in control cells at day 0 (beginning of the culture) and normalized to mRNA encoding hypoxanthine phosphoribosyl transferase, i.e., HPRT. (**C**) Localization of CD90 or CD105 (green) and nuclei (blue) in mADSCs after 72 h of culture, bar = 20 µm. (**D**) Analysis of the level of mRNAs encoding IL4R, IL13R, and CXCR7. Expression was related to the levels observed in control cells at day 0 (beginning of the culture) and normalized to mRNA encoding HPRT. (**E**) Localization of IL4R, IL13R, CXCR7, or CXCR4 (green) and nuclei (blue) in control mADSCs after 7 days of culture, bar = 20 µm. (**F**) In vitro migration assay—mADSCs were scratched from the culture dish and the area which was not invaded by migrating cells was measured and presented as the proportion (%) of the whole area photographed (0 h, 6 h, and 24 h). For each experimental group *n* ≥ 3. Data are presented as mean ± SD. Data have been analyzed using Student’s *t*-test (**A**); two-way ANOVA test (d-CXCR7, **F**); Kruskal-Wallis test (**B**,**D**—IL4R, IL13R). * *p* < 0.05; ** *p* < 0.01.

First, we analyzed mADSCs that were cultured in vitro in control medium or in the continuous presence of IL-4 or SDF-1. None of the treatments affected mADSC proliferation (Figure 1A). Analysis of the expression of mRNAs encoding CD90 and CD105, which are considered as the major markers of MSCs [19], showed that IL-4 significantly increased expression of CD90 in mADSCs (Figure 1B).

On the other hand, mRNA encoding CD105 was downregulated by IL-4 but not by SDF-1. Immunolocalization of both antigens did not reveal, however, significant differences between control ADSCs and those treated either with IL-4 or SDF-1 (Figure 1C). Analysis of the expression of IL-4 and SDF-1 receptors showed that ADSCs expressed mRNA encoding IL-4 type II receptor subunits, i.e., IL4R and IL13R (Figure 1D). In the case of SDF-1 receptors only mRNA encoding CXCR7 was detectable in mADSCs. However, we were able to detect both proteins, CXCR4 and CXCR7, as well as IL4R and IL13R using immunolocalization (Figure 1E). Knowing that both IL-4 and SDF-1 could influence cell migration we performed an in vitro scratch wound healing assay. ADSCs were cultured in control medium or in the presence of IL-4 or SDF-1. Once the culture reached confluency the scratch was made. The non-invaded area was assessed at 0, 6, and 24 h. Only SDF-1 treatment resulted in migration increase, as assessed 24 h after the scratch was made (Figure 1F).

**Figure 2:**
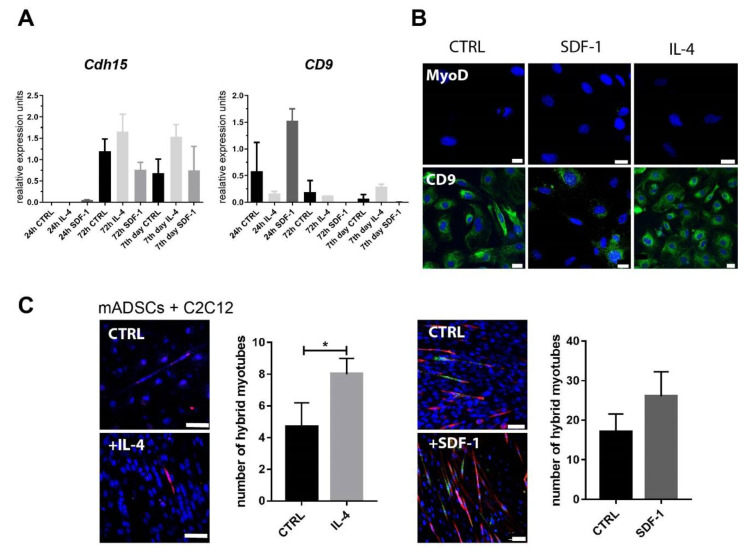
Characterization of mADSCs cultured under control conditions or in the presence of IL-4 or SDF-1. (**A**) Analysis of the level of mRNAs encoding M-cadherin (Cdh15) and CD9. Expression was related to the levels observed in control cells at day 0 (beginning of the culture) and normalized to mRNA encoding HPRT. (**B**) Localization of MyoD and CD9 (green) and nuclei (blue) in mADSCs after 7 days of culture, bar = 20 µm. (**C**) Co-culture between mADSCs and mouse C2C12 myoblasts in the presence of IL-4 or SDF-1. Images on the left present localization of Orange CMRA (red) and nuclei (blue). Images on the right present localization of GFP (green), skMyHC (red), and nuclei (blue), bar = 50 µm. Graphs present number of hybrid myotubes detected in five randomly chosen fields of view. For each experimental group *n* ≥ 3. Data are presented as mean ± SD. Data have been analyzed using Student’s *t*-test (**C**); Kruskal-Wallis test (**A**). * *p* < 0.05.

Next, we assessed if IL-4 or SDF-1 impact the ability of ADSCs to initiate myogenic differentiation in vitro. We assessed the expression of mRNA encoding MRFs, MYF5 and MYOD, and adhesion proteins M-cadherin and CD9. We did not detect mRNA encoding MYF-5 and MYOD, and cells positive for MYOD were not found. We were able to detect *Cdh15* (encoding M-cadherin), and *CD9* in mADSCs, however, treatment of cells with IL-4 or SDF-1 did not result in any significant differences in their expression (Figure 2A). The proportion of cells expressing CD9 was high, reaching 100% in control and IL-4-treated ADSCs and 52.6% in the case of those treated with SDF-1, at day 7 of culture (Figure 2B). Importantly, co-cultures of mADSCs with mouse C2C12 myoblasts showed that IL-4 but not SDF-1 significantly increased the ability of these cells to fuse with myoblasts and form hybrid myotubes (Figure 2C). 

### 3.2. Transplantation of IL-4- and SDF-1-Treated mADSCs into Regenerating Mouse Muscle

**Figure 3:**
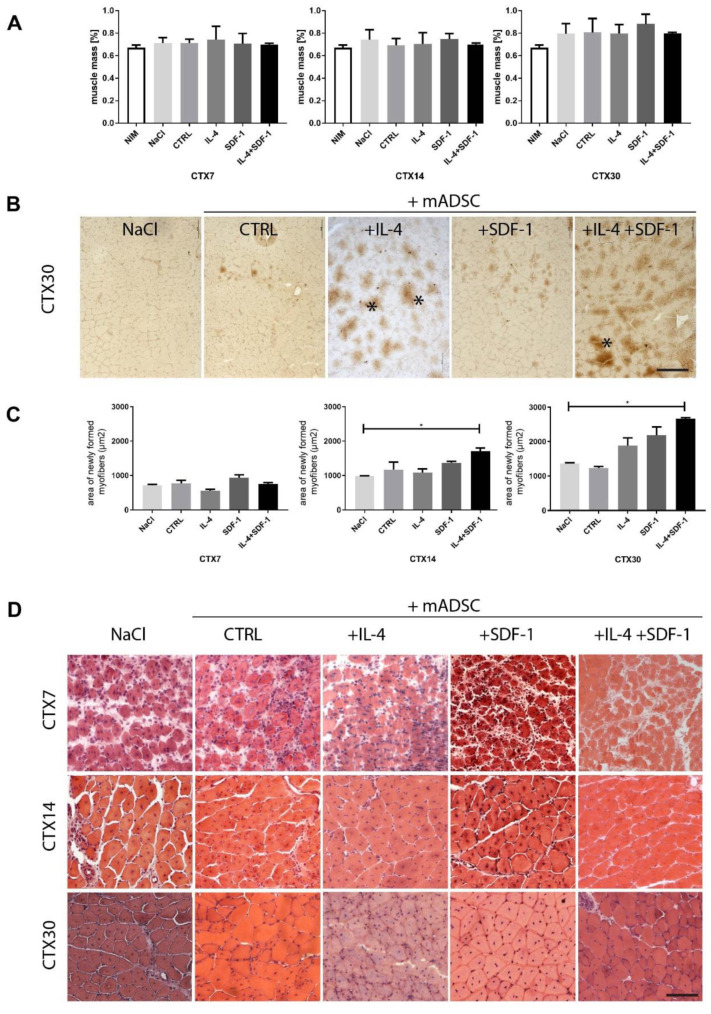
Regeneration of mouse skeletal muscles which received mADSCs - control or pretreated either with IL-4, SDF-1, or IL-4 and SDF-1. (**A**) Weight of intact, injured control and ADSC transplanted skeletal muscles related to the weight of the mouse, analyzed at day 7, 14, and 30 of regeneration. (**B**) Localization of mADSCs within the skeletal muscle at day 30 of regeneration using immunodetection of GFP (brown). Stars indicate skeletal myofibers generated with the participation of mADSCs, bar = 100 µm. (**C**) Area of newly formed myofibers within the regenerating muscle analyzed at day 7, 14, and 30 of regeneration depending on the injected solution. (**D**) Histological analysis of regenerating muscle injected with NaCl or with mADSCs and analyzed at day 7, 14, and 30 of regeneration, hematoxylin & eosin staining. CTX7 - day 7; CTX14 - day 14; CTX30 - day 30 of regeneration, Bar = 100 µm. For each experimental group *n* ≥ 3. Data have been analyzed using Kruskal-Wallis test. * *p* < 0.05.

**Figure 4:**
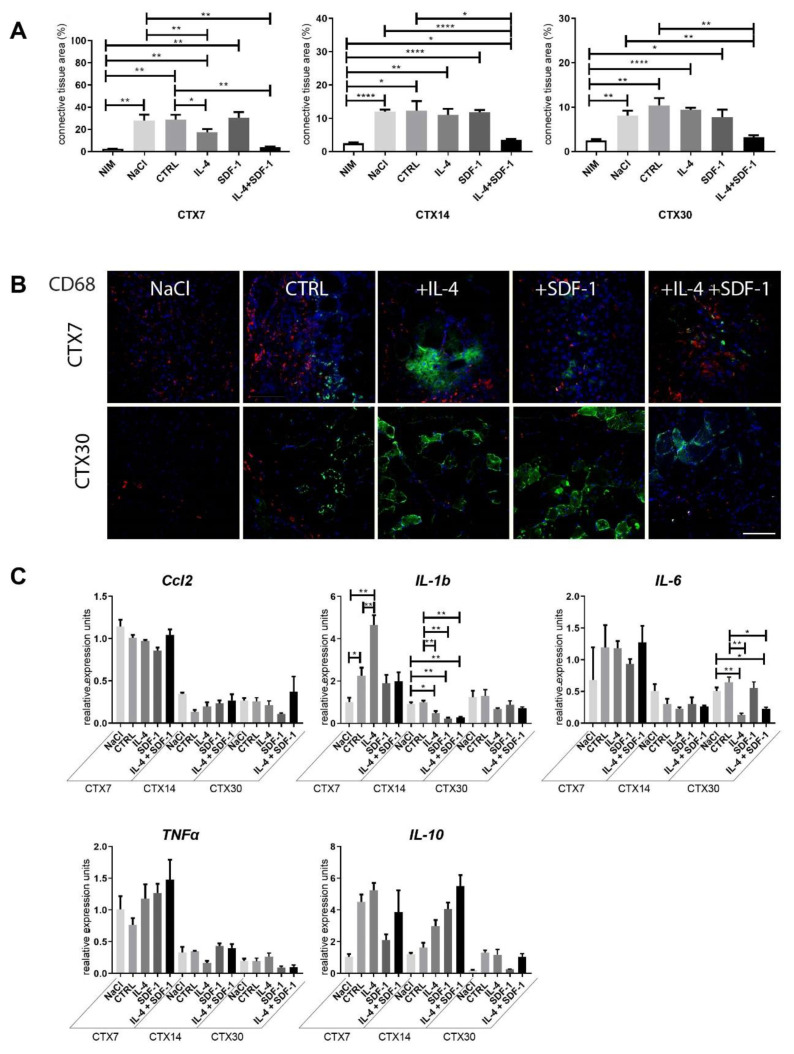
Connective tissue formation and immune response of mouse skeletal muscles which received NaCl, control mADSCs, or mADSCs pretreated with IL-4, SDF-1, or IL-4 and SDF-1. (**A**) Connective tissue area calculated from the histological sections (Masson’s Trichrome staining) analyzed at day 7, 14, and 30 of regeneration. (**B**) Localization of CD68+ cells and ADSCs in regenerating skeletal muscles assessed at day 7 and 30 of regeneration. CD68 (red), GFP (green), nuclei (blue), bar = 100 µm. (**C**) Expression of mRNAs encoding Ccl2, IL-1b, IL-6, TNFα, and IL-10, analyzed at day 7, 14, and 30 of regeneration. Expression was related to the mean expression level in control muscle analyzed at day 7 and normalized to mRNA encoding HPRT. CTX7 - day 7; CTX14 - day 14; CTX30 - day 30 of regeneration, For each experimental group *n* ≥ 3. Data are presented as mean ± SD. Data have been analyzed using two-way ANOVA test (a, c—IL-1b, IL-6); Kruskal-Wallis test (c-Ccl2, TNFα, IL-10). * *p* < 0.05; ** *p* < 0.01, *** *p* < 0.001, **** *p* < 0.0001.

IL-4 treatment resulted in increased ADSC ability to fuse with C2C12 cells (Figure 2C) Moreover, we showed that SDF-1 treatment improved mADSC migration (Figure 1F). Thus, we decided to test the ability of mADSCs treated either with IL-4 or SDF-1, as well as exposed to both factors, to improve mouse skeletal muscle regeneration. Mouse ADSCs used in this study were labelled using BacMam Transduction Control vector coding GFP, which allowed us to visualize the position of transplanted cells within the muscle. Gastrocnemius muscles of C57Bl6N mice were injured using CTX injection and either injected with NaCl or with cells pretreated with IL-4, SDF-1, or IL-4 and SDF-1 for 48 h. Muscles were then analyzed at day 7, 14, and 30 of regeneration. Comparison of the muscle weight, presented as the percentage of mouse weight, did not reveal any significant differences between all groups of analyzed muscles (Figure 3A). Histological analysis and immunolocalization of GFP, performed at day 30 of regeneration, documented that mADSCs not only survived within the regenerating muscles but also participated in the formation of new myofibers. Such phenomenon was the most profound in the case of the muscles which received IL-4 or IL-4 and SDF-1 treated mADSCs (Figure 3B). Further histological analyses revealed that the area of newly formed myofibers, i.e., the ones with centrally positioned nuclei, was significantly increased in the case of the skeletal muscles which received mADSCs pretreated with both factors, i.e., IL-4 and SDF-1 (Figure 3C,D). The improvement of skeletal muscle regeneration caused by the presence of mADSCs was also visible at the level of connective tissue formation. mADSCs treated with IL-4 or both, i.e., IL-4 and SDF-1, had significantly decreased connective tissue development at day 7. At day 14 and 30, a significantly lower connective tissue area was assessed in muscles injected with IL-4 and SDF-1 treated mADSCs, as compared to NaCl or untreated mADSC injected muscles (Figure 4A). Thus, the beneficial effect of mADSC transplantation and applied treatments was clearly visible at the histological level (Figure 3C,D and Figure 4A). 

The proper regeneration of skeletal muscles depends not only on the satellite cell function but also on the correct action of immune cells (for a review see [52,53]). Thus, we analyzed the presence of leukocytes characterized by the expression of CD45, pro-inflammatory M1 macrophages expressing CD68, and anti-inflammatory M2 macrophages expressing CD163 (for a review see [53,54]). At day 7 of regeneration, CD45+ cells were detectable in all analyzed muscle groups, but they disappear from the regenerating tissue by day 30 (data not shown). CD68+ cells were easily detected in all groups of muscles analyzed at day 7 of regeneration. At day 30 of regeneration, multiple M1 macrophages were still visible in muscles transplanted with control mADSCs, while they were virtually absent in muscles injected with IL-4, SDF-1, or IL-4 and SDF-1 treated cells (Figure 4B). No significant differences were found for CD163+ cells which were analyzed at day 7 and 30 (data not shown).

**Figure 5:**
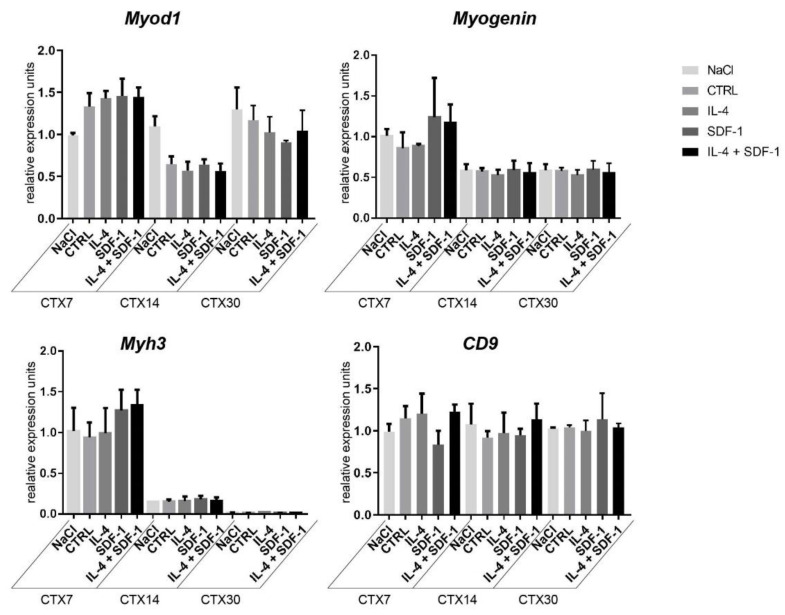
Expression of mRNA encoding myogenic factors in mouse skeletal muscles transplanted with mADSCs pretreated with either IL-4, SDF-1, or IL-4 and SDF-1. Analysis of the level of mRNAs encoding MYOD, MYOGENIN, MYH3, and CD9. Expression was related to the mean expression level in control muscle analyzed at day 7 and normalized to mRNA encoding HPRT. CTX7 - day 7; CTX14 - day 14; CTX30 - day 30 of regeneration, for each experimental group *n* ≥ 3. Data are presented as mean ± SD. Data have been analyzed using Kruskal-Wallis test.

To further investigate the influence of mADSCs on inflammatory state in regenerating muscles, we assessed the levels of mRNA encoding selected cytokines important for this process, i.e., CCL2 (C-C motif chemokine ligand 2), which is macrophage-produced cytokine responsible for attracting neutrophils necessary to remove cellular debris (for a review see [55]), IL-1b, IL-6, and TNFα that mediate inflammatory response and are produced, i.a., by infiltrating monocytes/macrophages [56,57,58], and IL-10—an anti-inflammatory cytokine that regulates changes in macrophage phenotype (for a review see, e.g., [59]). In the case of mRNAs encoding CCL2 or TNFα, their levels were high at day 7 after injury but then dropped with no significant differences found between all analyzed groups (Figure 4C). In the case of IL-1b, the initial increase observed 7 days after injury in muscles injected with IL-4 treated mADSCs was followed by a significant decrease by day 14, also in muscles injected with SDF-1 or IL-4 + SDF-1 treated mADSCs as compared to muscles injected with NaCl or non-treated mADSCs. IL-6 mRNA was significantly downregulated after transplantation of IL-4 or IL-4 and SDF-1 treated mADSCs in muscles analyzed at 30 day of regeneration. Expression of anti-inflammatory IL-10 was elevated in muscles receiving mADSCs, however, observed differences were not significant (Figure 4C). Thus, studied cells were able to effectively modulate the immune environment of regenerating muscle. Additionally, we assessed the expression of mRNAs encoding selected myogenic markers, i.e., MYOD, MYOGENIN, myosin heavy chain 3 (*Myh3*) and adhesion protein CD9, in regenerating muscles. We did not detect any significant differences between analyzed groups (Figure 5).

### 3.3. Human ADSC Response to IL-4 and SDF-1 Treatment In Vitro

**Figure 6:**
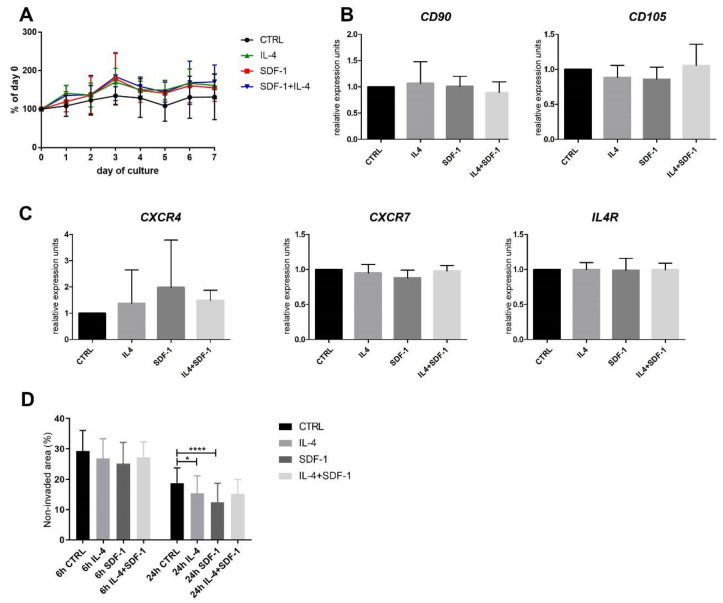
Characterization of hADSCs cultured under control conditions or in the presence of IL-4, SDF-1, or IL-4 and SDF-1. (**A**) Growth curves of ADSCs cultured for 7 days. (**B**) Analysis of the level of mRNAs encoding CD90 and CD105. Expression was related to the levels observed in control cells and normalized to mRNA encoding β-actin, i.e., ACTB. (**C**) Analysis of the level of mRNAs encoding CXCR4, CXCR7, and IL4R. Expression was related to the levels observed in control cells and normalized to mRNA encoding ACTB. (**D**) In vitro migration assay—hADSCs were scratched from the culture dish and the area which was not invaded by migrating cells was presented (6 h and 24 h) as the proportion (%) of the whole area photographed. For each experimental group *n* ≥ 3. Data are presented as mean ± SD. Data have been analyzed using Kruskal-Wallis test. * *p* < 0.05, **** *p* < 0.0001.

Human ADSCs were cultured in vitro in control medium or in medium supplemented with either IL-4, SDF-1, or IL-4 and SDF-1. None of the treatments affected hADSC proliferation, similarly as in the case of mADSCs (Figure 6A). Analysis of mRNAs encoding CD90, CD105, IL4R, CXCR4, and CXCR7 showed that none of the treatments changed the expression level of these transcripts (Figure 6B,C). On the other hand, IL-4 and SDF-1 influenced the migration of hADSCs: cells treated either with IL-4 or SDF-1 migrated significantly better than control cells, which was most profound at 24 h after the scratch was made (Figure 6D).

**Figure 7:**
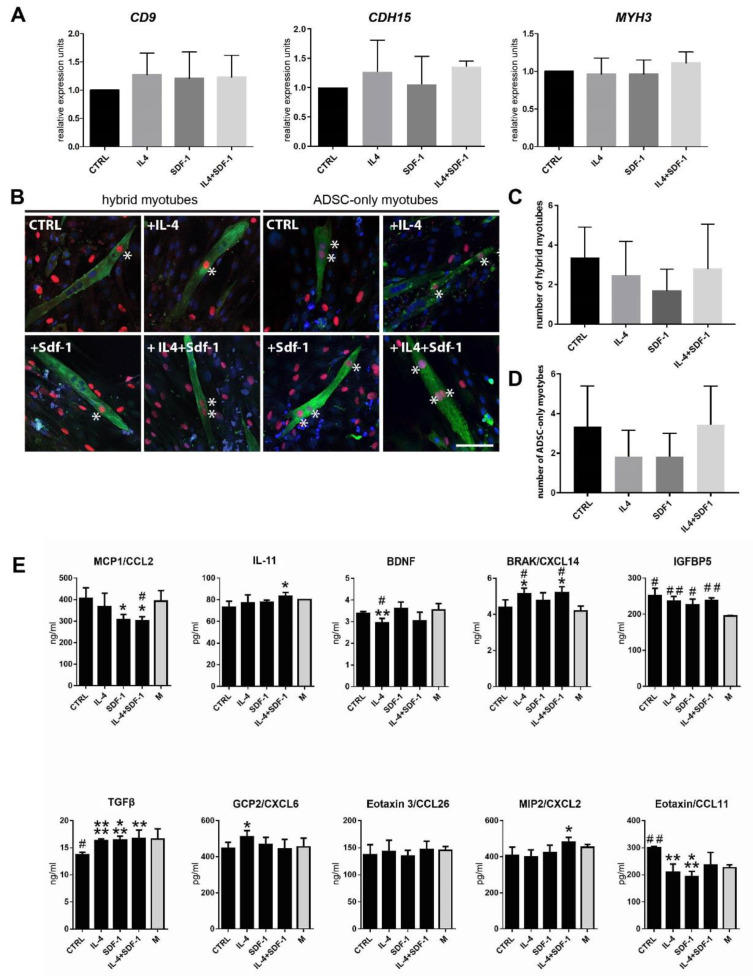
Characterization of hADSCs cultured under control conditions or in the presence of IL-4, SDF-1, or IL-4 and SDF-1. (**A**) Analysis of the level of mRNAs encoding CD9, M-cadherin (Cdh15), and MyH3. Expression was related to the levels observed in control cells and normalized to mRNA encoding ACTB. (**B**) Analysis of co-culture of hADSCs with mouse C2C12 myoblasts using immunolocalization of human nuclei (red), all nuclei (blue), and skeletal myosin (green), bar = 50 µm. (**C**) Number of myotubes formed in the co-culture between hADSCs and mouse C2C12 myoblasts. (**D**) Number of myotubes formed from hADSCs only. (**E**) Level of MCP1/CCL2, IL-11, BDNF, BRAK/CXCL14, IGFBP5, TGFβ, GCP2/CXCL6, Eotaxin 3/CCL26, MIP2/CXCL2, and Eotaxin/CCL11 was assayed using ELISA in medium in which hADSCs were cultured. For each experimental group *n* ≥ 3. Data are presented as mean ± SD. Data has been analyzed using Student’s t-test (**E**) or Kruskal-Wallis test (**A**,**C**,**D**). * *p* < 0.05; ** *p* < 0.01, *** *p* < 0.001, **** *p* < 0.0001, # *p* < 0.05, ## *p* < 0.01, ### *p* < 0.001, #### *p* < 0.0001. Asterisks depict the differences between the experimental samples and control samples; hashtags depict results related to the level in medium (M).

Next, we assessed how the IL-4 or SDF-1 or both of them impact the ability of hADSCs to initiate myogenic differentiation in vitro. We assessed the expression of mRNA encoding mesoderm marker, mesogenin (data not shown), and adhesion proteins CD9, M-cadherin (CDH-15), and V-CAM1 (data not shown), as well as MYH3. The levels of tested mRNAs did not significantly change after any treatment (Figure 7A).

Analysis of the hybrid myotube formation showed that none of the treatments had a beneficial effect on the ability of hADSCs to fuse with C2C12 myoblasts (Figure 7B,C). Surprisingly, hADSCs were able to fuse with each other and form myotubes without the participation of myoblasts (Figure 7D). Thus, in contrast to mADSCs, we did not notice significant impact of IL-4 at hybrid myotube formation, but similarly to mADSCs, we found SDF-1 and also IL-4 to be factors enhancing migration of hADSCs.

In the next step we investigated the potential immunomodulatory effect of hADSCs. First, we checked if the applied treatments modulated hADSC secretory activity in vitro (Figure 7E). Using an ELISA test we assayed the levels of the following secreted proteins: MCP1/CCL2, a pro-inflammatory protein monocyte chemoattractant (e.g., [60]); IL-11, an anti-inflammatory cytokine (e.g., [61]); BDNF, the factor whose level decreases during the development of inflammation (e.g., [62]); BRAK/CXCL14, a factor stimulating inflammatory cell migration and improving tissue regeneration [63]; IGFBP5, a factor improving tissue regeneration [64,65]; TGFβ, a growth factor which among other actions can stimulate M1-M2 macrophage transition [66]; GCP2/CXCL6; Eotaxin 3/CCL26, Eotaxin/CCL11, and MIP2/CXCL2, which act as granulocyte chemoattractants. Comparison of control and non-treated hADSCs with those treated either with IL-4, SDF-1, or IL-4 and SDF-1 showed that SDF-1 as well as IL-4 and SDF-1 treatment reduced the level of pro-inflammatory MCP1/CCL2. Treatment with both factors also increased the level of anti-inflammatory IL-11 as well as BRAK/CXCL14, which was also upregulated by IL-4 alone. Importantly, in all variants we observed an increase in the level of TGFβ. Next, hADSCs, control and treated, secreted IGFBP5, which was shown to have a beneficial effect on tissue regeneration. The level of eotaxin/CCL11 was downregulated (Figure 7E). Thus, we showed that combined IL-4 and SDF-1 treatment was the best choice to modulate the secretion of factors which could influence inflammation limitation and skeletal muscle regeneration improvement.

### 3.4. IL-4, SDF-1 or IL-4 and SDF-1 Impact at hADSC Transctiptome

**Figure 8:**
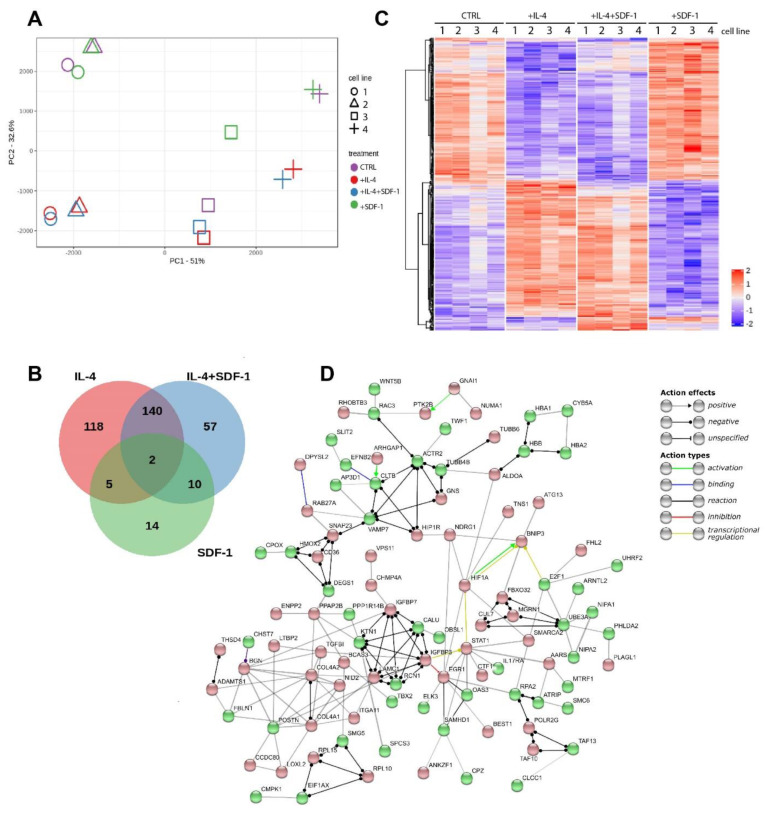
Global expression of mRNA in hADSCs cultured under control conditions or in the presence of IL-4, SDF-1, or IL-4 and SDF-1. (**A**) Principal component analysis (PCA) of four independent hADSCs. (**B**) Venn graph for 372 genes with a genome-wide significance (FDR < 0.005) from one-way ANOVA for the factor of treatment. Red circle shows genes affected by the IL-4 treatment, green by the SDF-1 treatment, and blue by the IL-4 and SDF-1 treatment. (**C**) Hierarchical clustering of transcripts regulated in response to treatment. RNA-seq results are shown as a heat map and include 372 genes with a genome-wide significance (FDR < 0.005) from one-way ANOVA for the factor of treatment. The intensity of the color is proportional to the standardized values (between −2 and 2) from each RNA-seq measurement, as indicated on the bar on the right of the heat map image. Hierarchical clustering was performed with R software using distance metrics correlation and the average linkage method. (**D**) Gene network for genes affected by the IL-4 and SDF-1 treatment. The network was created using STRING software and database. It shows protein–protein interaction. Up-regulated genes are marked in red and down-regulated in green.

Using mRNA sequencing we compared mRNA levels between four lines of hADSCs. Each line was subjected to the same treatment. Thus, we analyzed control, i.e., non-treated, as well as IL-4, SDF-1, or IL-4 and SDF-1 treated cells. Results of the sequencing have been deposited at SRA (accession number PRJNA623071). Principal component analysis (PCA) showed that two lines tended to group together (1 and 2). In each case, however, transcriptome profiles of IL-4 and IL-4 and SDF-1 treated hADSCs separated from those of control and SDF-1 treated cells (Figure 8A). The Venn graphs show the number of transcripts whose expression, depending on the applied treatment, is common or different from the control hADSCs, i.e., non-treated cells (Figure 8B, Table S2). Here, we see the highest number of transcripts was influenced in IL-4 or IL-4 and SDF-1 treated cells, but not in SDF-1 treated ones. The heat map presents 372 genes characterized by different expression levels (FDR < 0.005) (Figure 8C). As compared to control, i.e., untreated ADSCs, IL-4 changed the expression of 265 genes, SDF-1 changed the expression of 31 genes, and combined treatment with IL-4 and SDF-1 affected 209 genes. Interestingly, in the latter case, 140 of them were common also for IL-4 treatment, as compared to control cells (Figure 8B,C; all genes are listed in Table S2). In further analyses, using the Gene Ontology database, we focused on selected cellular processes showing how they were affected by separate or combined IL-4 and SDF-1 treatments (Figure 8D, Table S3).

We focused on the processes included in such categories, such as proliferation, migration, differentiation, and extracellular matrix reorganization, i.e., those ones related to tissue regeneration. We did not notice any massive changes in the expression of genes included in these categories after IL-4 and SDF-1 treatment (Table S3). Using the STRING tool we showed the interconnections between the genes regulating selected processes which were affected by the IL-4 and SDF-1 treatment. Among the upregulated genes were for example those whose products are involved in cell growth and proliferation (e.g., *IGFBP7*, *IGFBP3*, *TGFβ1*, *EGR1*). Next, we visualized upregulation of such genes as *ADAMTS1*, which is involved in the development of the inflammatory response and function of many tissues, and *ITGA11*, which encodes integrin α11 interacting with the extracellular matrix, as well as collagen genes *COL4A1*, *COL4A2*. Thus, the applied treatment impacted at the expression of many gene groups, including those which are involved in proliferation/differentiation processes. These analyses underlined, on the other hand, that despite the heterogeneity between analyzed hADSC lines (Figure 8A), they reacted similarly to the applied treatments. Knowing that, we turned to the in vivo analyses, allowing us to show the impact of hADSCs on skeletal muscle regeneration.

### 3.5. Transplantation of IL-4 and SDF-1 Treated hADSCs into Regenerating NOD/SCID Mouse Muscle

**Figure 9:**
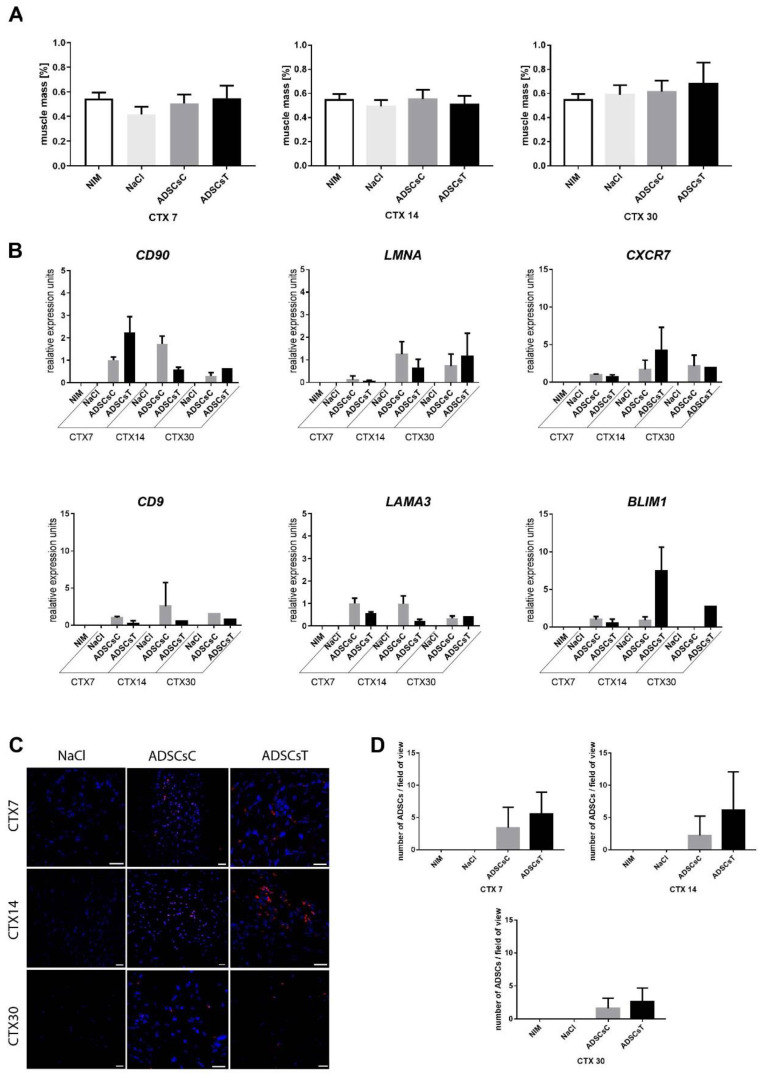
Regeneration of mouse skeletal muscles which received control hADSCs or hADSCs pretreated with IL-4 and SDF-1. (**A**) Weight of non-injured (NIM), injured control (NaCl), control hADSC (ADSCsC), and IL-4 and SDF-1 treated hADSC (ADSCsT) transplanted skeletal muscles related to the weight of the mouse, day 7, 14 and 30 of regeneration. (**B**) Expression of human mRNAs encoding CD90, LMNA, CXCR7, CD9, LAMA3, and BLIM1 in control and regenerating mouse muscles. Expression was normalized to mRNA encoding HPRT. (**C**) Localization of hADSCs within the skeletal muscle at day 7, 14, and 30 of regeneration using visualization of human nuclei (red) and all nuclei (blue), bar = 20 µm. (**D**) Number of hADSCs per field of view in injured control and ADSC transplanted skeletal muscles at day 7, 14, and 30 of regeneration. CTX7 - day 7; CTX14 - day 14; CTX30 - day 30 of regeneration, For each experimental group *n* ≥ 3. Data are presented as mean ± SD. Data have been analyzed using Kruskal-Wallis test.

**Figure 10:**
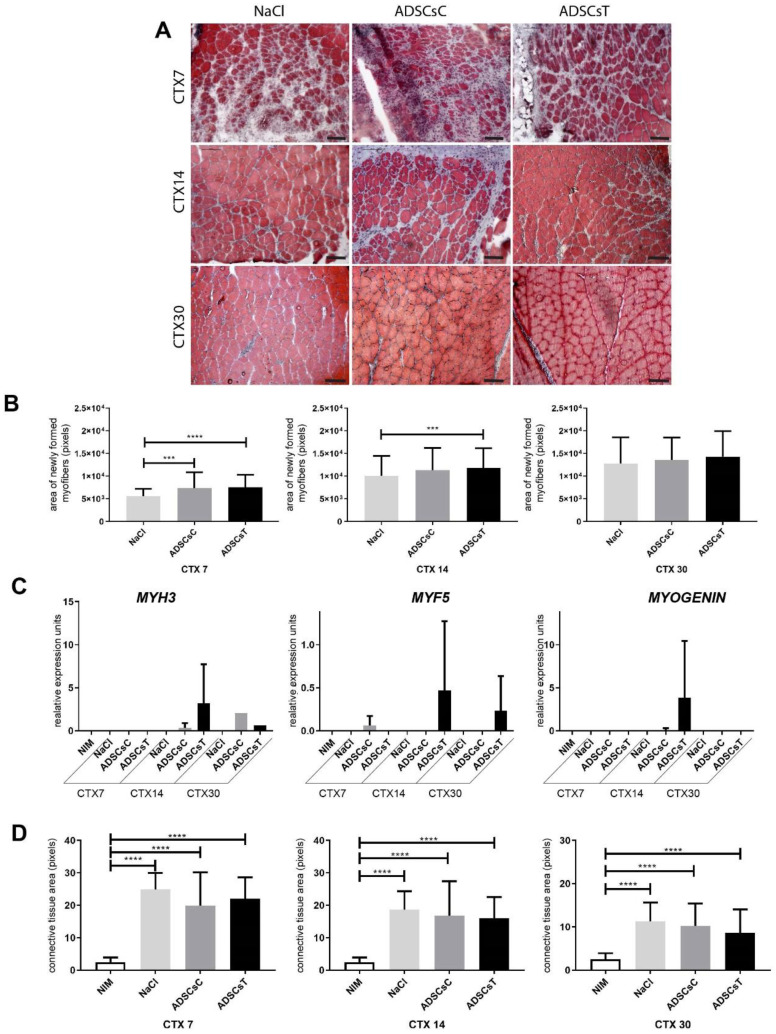
Regeneration of mouse skeletal muscles which received control hADSCs or hADSCs pretreated with IL-4 and SDF-1. (**A**) Morphology of regenerating mouse muscles at day 7, 14, and 30 of regeneration, Masson’s Trichrome staining, bar = 100 µm. (**B**) Area of newly formed myofibers within the regenerating muscle analyzed at day 7, 14, and 30 of regeneration. **(C)** Expression of human mRNAs encoding MYH3, MYF5, and MYOGENIN in control and regenerating muscles. Expression was normalized to mRNA encoding HPRT. (**D**) Connective tissue area calculated from the histological sections analyzed at day 7, 14, and 30 of regeneration. NIM—non-injured muscle, ADSCsC—muscle transplanted with control ADSCs, ADSCsT—muscle transplanted with treated ADSCs. CTX7 - day 7; CTX14 - day 14; CTX30 - day 30 of regeneration, For each experimental group *n* ≥ 3. Data are presented as mean ± SD. Data have been analyzed using Kruskal-Wallis test. * *p* < 0.05; ** *p* < 0.01, *** *p* < 0.001, **** *p* < 0.0001.

**Figure 11:**
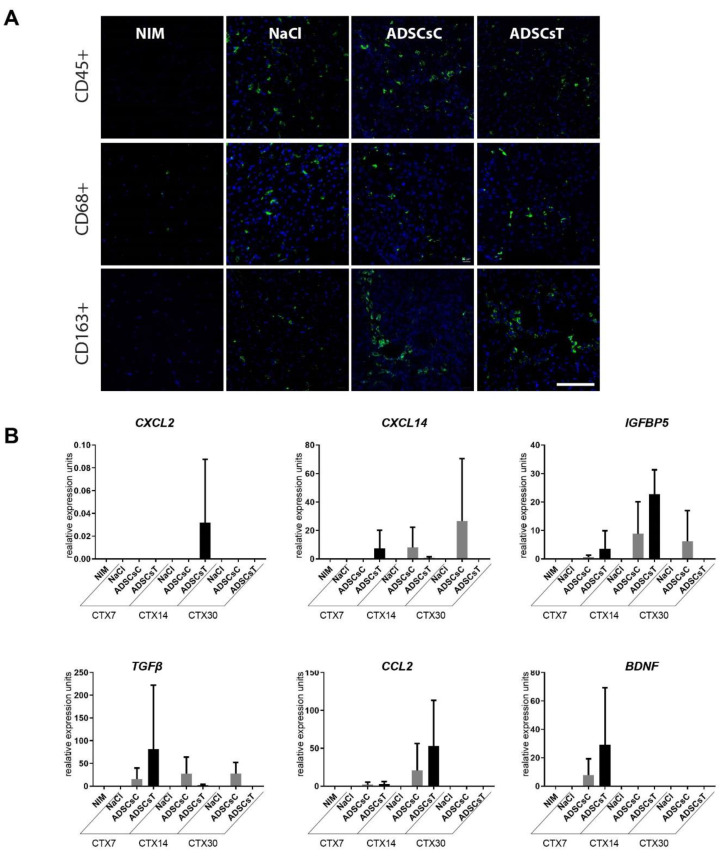
Regeneration of mouse skeletal muscles which received control hADSCs or hADSCs pretreated with IL-4 and SDF-1. (**A**) Localization of CD45+, CD68+, or CD163+ cells in regenerating skeletal muscles which received control ADSCs or ADSC pretreated with IL-4 and SDF-1 assessed at day 7 of regeneration. CD45+, CD68+, and CD163+ cells (green), nuclei (blue), bar = 20 µm. (**B**) Expression of mRNAs encoding human CXCL2, CXCL14, IGFBP5, TGFb, CCL2, and BNDF. Expression was normalized to mRNA encoding HPRT. For each experimental group *n* ≥ 3. Data are presented as mean ± SD. Data have been analyzed using Kruskal-Wallis test.

We showed that IL-4 as well as SDF-1 improved hADSC migration (Figure 6D) and that combined treatment with IL-4 and SDF-1 was beneficial in terms of the secretion of factors potentially facilitating proper regeneration of skeletal muscle (Figure 7E). Thus, in the next step we transplanted hADSCs pretreated for 48 h with both IL-4 and SDF-1 to gastrocnemius muscles of NOD/SCID mice, previously injured by CTX injection. Control muscles were injected with NaCl or with non-treated hADSCs. Muscles were isolated and analyzed at day 7, 14, and 30 of regeneration. Comparison of the muscle weight, presented as the percentage of mouse weight, showed that transplantation of hADSCs did not significantly affect muscle mass (Figure 9A). Next, we assessed the level of human mRNAs encoding stem cell marker CD90, nuclear lamin A (hLMNA), CXCR7, CD9, laminin a3 (LAMA3), and BLIM1 in regenerating mouse muscles (Figure 9B). All analyzed human transcripts were expressed only in the muscles transplanted with hADSCs. Immunolocalization of human nuclear antigen within muscle sections, performed at day 7, 14, and 30 of regeneration, documented that hADSCs were indeed present within the regenerating muscles (Figure 9C,D). Importantly, the areas of newly formed myofibers, i.e., the ones with centrally positioned nuclei, were significantly increased in skeletal muscles transplanted with either control or pretreated hADSCs at day 7 of regeneration. At day 14 such difference was observed only in the case of hADSCs pretreated with IL-4 and SDF-1 (Figure 10A,B). Moreover, the transcripts encoding myogenic markers, *MYF5, MYOGENIN,* and *MYH3*, of human origin, however at the low level, were found only in muscles injected with treated hADSCs (Figure 10C). Importantly, the presence of hADSCs did not induce excessive deposition of ECM proteins as revealed by connective tissue area analysis. We did not observe any significant differences between regenerating muscles, regardless of the treatment applied (Figure 10D). Next, we followed the immune cells within the NOD/SCID mice regenerating muscles, which, as we previously showed, produce reduced numbers of M1 and M2 macrophages [67]. We did not notice any significant differences in the CD45+, CD68+, and CD163+ cell infiltration between control, i.e., NaCl injected muscles, and muscles injected with control or pretreated hADSCs (Figure 11A).

Next, we looked at the expression of human mRNAs encoding CXCL2, CXCL14 (BRAK), IGFBP5, TGFβ, CXCL2 (MIP2), and BNDF (Figure 11B). We did detect the expression of all the abovementioned transcripts in hADSCs, documenting that they impose an immunomodulatory effect on the tissue they were transplanted in, acting as factors positively modulating skeletal muscle regeneration.

Summarizing, testing two models, mouse and human, we documented the differences between the ADSCs of these species. Thus, we confirmed that results obtained using one model cannot be simply extrapolated to the other. Importantly, we showed that IL-4, SDF-1, and a combination of both factors can be used to enhance the potential of ADSCs to support skeletal muscle regeneration. Using these factors one can increase the ability of ADSCs to fuse, as shown for mouse cells, boost their migration and immunomodulatory potential, and significantly improve skeletal muscle regeneration.

## 4. Discussion

Adipose tissue-derived stromal cells (ADSCs) originating from two species, i.e., mouse and human, were analyzed in the current study. Behind our choice were multiple lines of evidence showing that these cells possess features that document their therapeutic potential. On the basis of their characteristics, these cells are considered as mesenchymal stromal cells (MSCs), which can be isolated from such sources as bone marrow, adipose tissue, skeletal muscle, and when properly treated can differentiate to adipose tissues but also bone, cartilage, or tendons ([18], for a review see [68]). Moreover, many reports show that their multipotency can be also manifested by an ability to differentiate into such cell types as myoblasts [69] or even those of other than mesodermal origin, e.g., neurons originating from ectoderm [70,71]. Our previous study, during which we analyzed human MSCs isolated from umbilical cord connective tissue, showed that in vitro they were able to form hybrid myotubes with C2C12 cells and to colonize skeletal muscle tissue and improve its regeneration [46]. However, the efficiency of MSC differentiation into skeletal myoblasts is still challenging, as also shown in other studies documenting the ability of MSCs to participate in new myofiber formation, however, with very low frequency (e.g., [72,73,74]). As far as ADSCs are concerned, they were shown to be able to participate in the regeneration of skeletal muscles of *mdx* mice [75]. However, even if MSCs were not involved in the generation of myofibers, their presence in the muscle was shown by us [76] and also others (e.g., [77,78,79]) to be beneficial for its regeneration due to their immunomodulatory function.

In the current work, we performed a variety of in vitro and in vivo analyses allowing mouse and human ADSCs and their responses to two factors known to be involved in myogenic differentiation and cell migration to be characterized and compared. Interleukin-4 (IL-4) was previously shown to regulate formation of myotubes [35] and enhance migration of myogenic precursor cells [37], as well as epithelial cells [80]. Stromal derived factor-1 (SDF-1, CXCL12) is an effective chemoattractant of MSCs and also a factor influencing cell survival and differentiation (e.g., [81]). This property was also extensively studied by us, proving that SDF-1 can mobilize precursor cells to the injured skeletal muscle [44] and increase the expression of CD9—tetraspanin crucial for skeletal myoblast fusion [50]. Importantly, it was shown that SDF-1 protects ADSCs from apoptosis, which enhances the ability of these cells to facilitate wound healing [82]. Thus, by using these two factors, alone or in combination, we hoped to modulate ADSC differentiation and their ability to support skeletal muscle regeneration. Our in vitro studies showed that IL-4 or SDF-1 did not have any impact at the proliferation of mouse and human cells. Importantly, both analyzed factors increased the migration ability of hADSCs, while mADSCs reacted only to SDF-1 treatment. In addition, IL-4 increased the proportion of hybrid myotubes between mADSCs and mouse C2C12 myoblasts; human cells did not present such a reaction. On the other hand, only hADSCs were able to form myotubes without participation of C2C12 myoblasts, i.e., the myotubes containing exclusively human nuclei—such myotubes were never observed in the co-cultures between mADSCs and C2C12 cells. Neither mouse nor human cells changed the expression of analyzed adhesion proteins in the response to any treatment. We did not detect any significant upregulation in the expression of mRNAs encoding myogenic regulatory factors nor muscle specific structural proteins in in vitro cultured ADSCs. Our transcriptome analysis focusing at hADSCs only proved that IL-4, SDF-1, and also a combination of both factors have significant effect on the transcriptome changes. In this case, we also proved that hADSCs derived from different patients, not as in the case of mouse from inbred strains of laboratory animals, differ between each other. Such differences were reported before [30]. However, they did not affect the differentiation potential of analyzed hADSCs.

In vivo analyses of mouse and human ADSCs proved that regardless of origin these cells were able to improve skeletal muscle regeneration. The major mode of action was attributed to their immunomodulatory ability. Importantly, it was enhanced by applied treatments, and combination of IL-4 and SDF-1 had the most profound effect. Thus, we observed a decrease in the presence of pro-inflammatory macrophages and beneficial changes in the expression of the factors improving regeneration, as we first showed for hADSCs in vitro (ELISA) and then in vivo, analyzing regenerating muscles. The modulation of hADSC secretory activity was manifested both in vitro in the expression of *CCL2, IL-11, CXCL14, IGFBP5, TGFβ*, and *CCL11* and in vivo in the expression of *CCL2, CXCL14, IGFBP5,* and *TGFβ*. Furthermore, others reported that ADSCs secrete a large number of factors, such as HGF, TGFβ, TNFα, SDF-1, interleukins, and many others, documenting their paracrine effect [83,84,85,86]. Our study documents that in the case of hADSC their secretory action might be the major one that improves regeneration. Moreover, we noticed that transplantation of ADSCs reduced the development of fibrosis. On the other hand, in case of hADSCs we observed upregulation of collagen 4, which is the component of basal lamina, which again suggests that the presence of these cells improves re-formation of the muscle fibers. Moreover, in the case of mADSCs we also observed their participation in myofiber formation, which was significantly enhanced by IL-4 or combined IL-4 and SDF-1 treatment. Recently, we showed that also other factors, such as silencing of TGFβ signaling, combined with mADSCs can significantly improve muscle repair—again also by modulating immune response [76]. Thus, our results support the thesis that application of ADSCs could be beneficial for skeletal muscle tissue repair.

## Supplementary Materials

The following are available online at https://www.mdpi.com/2073-4409/9/6/1479/s1, Table S1: Taqman assays used in qPCR analyses. Table S2: List of genes whose expression was changed as a result of IL-4, SDF-1, or IL-4 and SDF-1 treatment and which were visualized in Venn graphs (Figure 8B). Table S3: Selected molecular and cellular functions predicted to differ between IL-4 and SDF-1 and control, i.e., non-treated hADSCs (according to Gene Ontology and GO Annotations).
