# Supplementary material for: Beneficial Effect of IL-4 and SDF-1 on Myogenic Potential of Mouse and Human Adipose Tissue-Derived Stromal Cells
Source: Cells. 2020 Jun 17;9(6):1479. doi: 10.3390/cells9061479 (PMC7349575; doi:10.3390/cells9061479)
Supplement: Supplementary file 1 [file cells-09-01479-s001.pdf]

Beneficial Effect of IL-4 and SDF-1 on Myogenic Potential of Mouse and Human Adipose Tissue-Derived Stromal Cells

Karolina Archacka [1,†], Joanna Bem [1,†], Edyta Brzoska [1,†], Areta M. Czerwinska [1,†], Iwona Grabowska [1], Paulina Kasprzycka [1], Dzesika Hoinkis [2], Katarzyna Siennicka [3], Zygmunt Pojda [3], Patrycja Bernas [1], Robert Binkowski [1], Kinga Jastrzebska [1], Aleksandra Kupiec [1], Malgorzata Malesza [1], Emilia Michalczewska [1], Marta Soszynska [1], Katarzyna Ilach [1], Wladyslawa Streminska [1] and Maria A. Ciemerych [1,*]

[1] Department of Cytology, Institute of Developmental Biology and Biomedical Sciences, Faculty of Biology, University of Warsaw, Ilji Miecznikowa 1, 02-096 Warsaw, Poland; kczaja@biol.uw.edu.pl (K.A.); j.bem@cent.uw.edu.pl (J.B.); edbrzoska@biol.uw.edu.pl (E.B.); areta@biol.uw.edu.pl (A.M.C.); igrabowska@biol.uw.edu.pl (I.G.); p.kasprzycka@biol.uw.edu.pl (P.K.); p.bernas@student.uw.edu.pl (P.B.); r.binkowski@student.uw.edu.pl (R.B.); kinga.jastrzebska@student.uw.edu.pl (K.J.); aleksandra.kupiec@student.uw.edu.pl (A.K.); malgorzata.malesza@student.uw.edu.pl (M.M.); emiliachudek@student.uw.edu.pl (E.M.); m.soszynska4@student.uw.edu.pl (M.S.), kilach@biol.uw.edu.pl (K.I.); krymar@biol.uw.edu.pl (W.S.)

[2] Intelliseq Ltd., Stanisława Konarskiego 42/13, 30-046 Krakow, Poland; dzesika.hoinkis@intelliseq.pl

[3] Department of Regenerative Medicine, Maria Sklodowska-Curie National Research Institute of Oncology, W.K. Roentgena 5, 02-781 Warsaw, Poland; katarzyna.siennicka@pib-nio.pl (K.S.); zygmunt.pojda@coi.pl (Z.P.)

* Correspondence: ciemerych@biol.uw.edu.pl; Tel.: +48-22-55-42-216

† These authors contributed equally.

Table 1. Taqman assays used in qPCR analyzes.

Species	Gene	Taqman Assay No.	Species	Gene	Taqman Assay No.
MOUSE	*Cd90*	Mm00493682_g1	HUMAN	*CD90*	Hs00264235_m1
	Cd105	Mm00468256_m1		*CD105*	Hs00923996_m1
	IL4R	Mm01275139_m1		*IL4R*	Hs00965056_m1
	IL13R	Mm00446726_m1		*CXCR4*	Hs00976734_m1
	Cxcr7	Mm02619632_s1		*CXCR7*	Hs00664172_s1
	Myod	Mm00440387_m1		*CD9*	Hs01124022_m1
	Myf5	Mm00435125_m1		*CDH15*	Hs00170504_m1
	Cdh15	Mm00483191_m1		*DESMIN*	Hs00157258_m1
	Myh3	Mm01319006_g1		*MYF5*	Hs00271574_m1
	Myogenin	Mm00446194_m1		*MYOGENIN*	Hs01072232_m1
	Cd9	Mm00514275_g1		*MYH3*	Hs01074230_m1
	Ccl2	Mm00441242_m1		*LMNA*	Hs00153462_m1
	IL-1b	Mm00434228_m1		*LAMA3*	Hs00165042_m1
	IL-6	Mm00446190_m1		*BLIM*	Hs01046520_m1
	Tnf-α	Mm00443258_m1		*CXCL2*	Hs00601957_m1
	Il=L-10	Mm01288386_m1		*CXCL14*	Hs01557413_m1
	Hprt	Mm00446968_m1		*IGFBP5*	Hs00181213_m1
				TGFB	Hs00998133_m1
				CCL2	Hs00234140_m1
				BDNF	Hs01055414_m1
				ACTB	Hs01060665_g1
				HPRT	Hs02800695_m1

Table 2. List of genes which expression was changed as a result of IL-4, SDF-1 or IL-4 and SDF-1 treatment and which were visualized in Venn graphs (Figure 8B).

Control Versus Selected Treatments	Number of Genes	Gene List
IL-4	265	JARID2, CYB561, MBTD1, ZRANB1, NCAPH2, VRK2, ARNTL2, ANK1, CHPF2, BOD1L1, TNC, GOPC, OGFR, AP3D1, RASSF1, ATP11A, ABCC9, TCF3, C19orf10, KIFAP3, FBLN1, SMARCA2, CPOX, CHMP2B, NID2, AARS, SEL1L3, CCDC80, PSME1, SNAP23, DPYSL2, KCNK6, TTLL12, HIF1A, C20orf24, SLC17A9, SAMHD1, E2F1, SRPX2, PLA2G15, HMOX2, LACTB, VPS18, NDRG1, BNIP3L, PPP6R1, DHX40, MED13, LRRC59, SLC9A3R1, TMEM104, NDUFC1, CPZ, KLF3, CCDC86, TMEM109, ELK3, WNT5B, SH2B3, OAS3, ST8SIA1, SENP6, ARRDC3, SUB1, TARS, CRBN, MAPKAPK3, AUP1, DOK1, STAT1, GLS, FHL2, PASK, PLEKHB2, COX7A2L, RPA2, PHF3, PLAGL1, KLF12, GTF3C3, KLF9, TMEM214, GPAM, WDR11, EGR1, GLT8D2, CCDC53, PTK2B, CLCC1, FAM126A, HJURP, OBSL1, FAM210B, USP9X, EFNB2, NCLN, GNAI1, MPST, CALU, SPCS3, TRPM4, HELZ2, PAK4, SH3BGRL, TRAF7, CLUH, MORC4, BTG1, TMEM106C, SLC38A2, BTF3L4, FADS2, COL4A2, NREP, OSTF1, CD36, PHF21A, PRADC1, GNS, LAMC1, ENPP2, NUMA1, LRRC32, RSL24D1, CIR1, CCNG2, NIPA2, TSPAN3, NCOA2, DEF8, ABCA8, BCAS3, PTRH2, COL6A2, AKT1, EMP3, CARM1, FHAD1, PIGK, DCAF6, ADAM15, DEGS1, SFXN5, FBLN7, ZC3H8, OSBPL10, SLIT2, RPS3A, CBR4, SRD5A1, PNRC1, IGFBP3, SLC12A9, SLC16A2, CHST7, MFHAS1, SNX30, CTF1, HNMT, C11orf57, JMY, RMND5A, KCTD15, ANKH, ENAH, ADAMTS1, SLC16A1, DEPTOR, PHKG2, TMEM171, NBL1, C19orf47, MGAT4B, PPAP2B, AGL, ZNF281, CGGBP1, IGFBP7, ANKZF1, ATRIP, RHOBTB3, ANKRA2, C7orf60, TMEM65, DACT1, HTRA1, TAF10, PPFIBP2, RPL27A, PIP4K2C, SCG5, PPP2R3B, PPP1R14A, SPINT2, YIF1B, FAM57A, KCTD5, BEST1, POLR2G, STIP1, GDNF, GPRIN1, RAC3, PUSL1, NIPA1, DCLK2, C11orf24, ATP6V0E2, CANT1, RPS7, PPP1R3B, MAP3K11, PPP1R14B, LRFN4, SLC19A1, C1QTNF1, C16orf91, RGMB, RPL15, ARHGAP1, ATG13, CLTB, TUBB6, DIRAS1, C18orf32, IL17RA, ARL6IP6, RIC8A, RPP25, MPI, MTURN, PHLDA2, MFSD5, SETD3, TMED9, RPS27L, PURA, ZNF397, COL4A1, THSD4, TUBB4B, NBR1, ZNF548, FAM180A, EIF4BP6, ATAD3A, MPZL1, MFAP3L, SMG5, TGM2, BMPR2, REPIN1, MIR503HG, ERCC6, RPL41, MIF, STARD4-AS1, PGAM5, GS1-251I9.4, CHMP4A, RP11-166D19.1, RP11-473I1.10, RPL10, MUSTN1, RP11-572C15.6
IL-4+SDF-1	209	JARID2, ZRANB1, VRK2, ARNTL2, ANK1, CUL7, RCN1, AP3D1, RASSF1, ABCC9, RAB27A, PHRF1, TCF3, TEAD2, FBLN1, TNS1, SMARCA2, CPOX, NID2, AARS, CCDC80, PSME1, SNAP23, DPYSL2, HIF1A, TRPC4AP, C20orf24, SAMHD1, E2F1, USP11, SRPX2, MGRN1, PLA2G15, HMOX2, LACTB, NDRG1, ERI1, DHX40, SLC9A3R1, NDUFC1, CPZ, MTCH2, TMEM109, ELK3, WNT5B, SH2B3, OAS3, ST8SIA1, RWDD1, C5orf15, UBE3A, REEP6, AUP1, DOK1, LANCL1, STAT1, FHL2, PLEKHB2, ICMT, RPA2, PLAGL1, KLF12, LTBP2, MTRF1, TGFBI, EGR1, GLT8D2, CCDC53, PTK2B, TBX2, CLCC1, FAM126A, GLIPR2, HJURP, MXD4, OBSL1, VAMP7, EFNB2, KTN1, GNAI1, MPST, CALU, SPCS3, HIP1R, SYNE1, SH3BGRL, CLUH, POMP, POSTN, MORC4, LOXL2, TMEM106C, SLC38A2, COL4A2, NREP, OSTF1, CD36,

		GNS, LAMC1, ENPP2, DSCC1, NUMA1, ITGA11, ACTR2, FGF5, NIPA2, TSPAN3, ZFHX3, BCAS3, PTRH2, SLC39A6, FHAD1, DEGS1, OSBPL10, SLIT2, SRD5A1, IGFBP3, SLC12A9, C7orf55-LUC7L2, CHST7, TACC1, UHRF2, SNX30, HSD17B12, ALDOA, CTF1, TWF1, PTPRK, RMND5A, KCTD15, ADAMTS1, DEPTOR, FBXO32, TMEM171, VPS11, MGAT4B, SYVN1, CMPK1, PPAP2B, SMC6, IGFBP7, ANKZF1, ATRIP, RHOBTB3, ANKRA2, C7orf60, ZNF367, DACT1, TAF10, CYB5A, C11orf74, PPFIBP2, PIP4K2C, SCG5, PPP2R3B, PPP1R14A, BEST1, POLR2G, GPRIN1, RAC3, NIPA1, DCLK2, C11orf24, ATP6V0E2, TVP23B, PPP1R3B, PPP1R14B, LRFN4, EIF1AX, C16orf91, RGMB, RPL15, ARHGAP1, ATG13, CLTB, TUBB6, BNIP3, IL17RA, ARL6IP6, CTD-2192J16.15, RPP25, MPI, SSC5D, PHLDA2, SETD3, COL4A1, THSD4, TUBB4B, HBA2, FAM111B, FAM180A, TAF13, MPZL1, PAPSS2, MFAP3L, SMG5, HBA1, MIR503HG, MSL3P1, ATF6B, hsa-mir-6723, MIF, HBB, SOCS2-AS1, CHMP4A, RP11-166D19.1, RPL10, BGN, RP11-572C15.6
SDF-1	31	DNAJC25, NCKAP1, IFT80, CECR5, NUCB2, MGRN1, UBE2W, DHX40, DAP, TARS, UBE3A, PTBP3, MTRF1, MXD4, VAMP7, BTF3L4, FADS2, KIAA1033, DEF8, RCN3, UBA3, TWF1, CCDC122, ABCA5, SYVN1, SMC6, FAM57A, HSPBAP1, GPRIN1, MSL3P1, hsa-mir-6723
IL-4 and IL-4+SDF-1	142	JARID2, ZRANB1, VRK2, ARNTL2, ANK1, AP3D1, RASSF1, ABCC9, TCF3, FBLN1, SMARCA2, CPOX, NID2, AARS, CCDC80, PSME1, SNAP23, DPYSL2, HIF1A, C20orf24, SAMHD1, E2F1, SRPX2, PLA2G15, HMOX2, LACTB, NDRG1, DHX40, SLC9A3R1, NDUFC1, CPZ, TMEM109, ELK3, WNT5B, SH2B3, OAS3, ST8SIA1, AUP1, DOK1, STAT1, FHL2, PLEKHB2, RPA2, PLAGL1, KLF12, EGR1, GLT8D2, CCDC53, PTK2B, CLCC1, FAM126A, HJURP, OBSL1, EFNB2, GNAI1, MPST, CALU, SPCS3, SH3BGRL, CLUH, MORC4, TMEM106C, SLC38A2, COL4A2, NREP, OSTF1, CD36, GNS, LAMC1, ENPP2, NUMA1, NIPA2, TSPAN3, BCAS3, PTRH2, FHAD1, DEGS1, OSBPL10, SLIT2, SRD5A1, IGFBP3, SLC12A9, CHST7, SNX30, CTF1, RMND5A, KCTD15, ADAMTS1, DEPTOR, TMEM171, MGAT4B, PPAP2B, IGFBP7, ANKZF1, ATRIP, RHOBTB3, ANKRA2, C7orf60, DACT1, TAF10, PPFIBP2, PIP4K2C, SCG5, PPP2R3B, PPP1R14A, BEST1, POLR2G, GPRIN1, RAC3, NIPA1, DCLK2, C11orf24, ATP6V0E2, PPP1R3B, PPP1R14B, LRFN4, C16orf91, RGMB, RPL15, ARHGAP1, ATG13, CLTB, TUBB6, IL17RA, ARL6IP6, RPP25, MPI, PHLDA2, SETD3, COL4A1, THSD4, TUBB4B, FAM180A, MPZL1, MFAP3L, SMG5, MIR503HG, MIF, CHMP4A, RP11-166D19.1, RPL10, RP11-572C15.6
IL-4+SDF-1 and SDF-1	12	MGRN1, DHX40, UBE3A, MTRF1, MXD4, VAMP7, TWF1, SYVN1, SMC6, GPRIN1, MSL3P1, hsa-mir-6723
IL-4 and SDF-1	7	DHX40, TARS, BTF3L4, FADS2, DEF8, FAM57A, GPRIN1
IL-4 and IL-4+SDF-1 and SDF-1	2	DHX40, GPRIN1
IL-4 only	118	CYB561, MBTD1, NCAPH2, CHPF2, BOD1L1, TNC, GOPC, OGFR, ATP11A, C19orf10, KIFAP3, CHMP2B, SEL1L3, KCNK6, TTLL12, SLC17A9, VPS18, BNIP3L, PPP6R1, MED13, LRRC59, TMEM104, KLF3, CCDC86, SENP6, ARRDC3, SUB1, CRBN, MAPKAPK3, GLS, PASK, COX7A2L, PHF3, GTF3C3, KLF9, TMEM214, GPAM, WDR11, FAM210B, USP9X, NCLN, TRPM4, HELZ2, PAK4, TRAF7, BTG1, PHF21A, PRADC1,

		LRRC32, RSL24D1, CIR1, CCNG2, NCOA2, ABCA8, COL6A2, AKT1, EMP3, CARM1, PIGK, DCAF6, ADAM15, SFXN5, FBLN7, ZC3H8, RPS3A, CBR4, PNRC1, SLC16A2, MFHAS1, HNMT, C11orf57, JMY, ANKH, ENAH, SLC16A1, PHKG2, NBL1, C19orf47, AGL, ZNF281, CGGBP1, TMEM65, HTRA1, RPL27A, SPINT2, YIF1B, KCTD5, STIP1, GDNF, PUSL1, CANT1, RPS7, MAP3K11, SLC19A1, C1QTNF1, DIRAS1, C18orf32, RIC8A, MTURN, MFSD5, TMED9, RPS27L, PURA, ZNF397, NBR1, ZNF548, EIF4BP6, ATAD3A, TGM2, BMPR2, REPIN1, ERCC6, RPL41, STARD4-AS1, PGAM5, GS1-251I9.4, RP11-473I1.10, MUSTN1
IL-4+SDF-1 only	57	CUL7, RCN1, RAB27A, PHRF1, TEAD2, TNS1, TRPC4AP, USP11, ERI1, MTCH2, RWDD1, C5orf15, REEP6, LANCL1, ICMT, LTBP2, TGFBI, TBX2, GLIPR2, KTN1, HIP1R, SYNE1, POMP, POSTN, LOXL2, DSCC1, ITGA11, ACTR2, FGF5, ZFHX3, SLC39A6, C7orf55-LUC7L2, TACC1, UHRF2, HSD17B12, ALDOA, PTPRK, FBXO32, VPS11, CMPK1, ZNF367, CYB5A, C11orf74, TVP23B, EIF1AX, BNIP3, CTD-2192J16.15, SSC5D, HBA2, FAM111B, TAF13, PAPSS2, HBA1, ATF6B, HBB, SOCS2-AS1, BGN
SDF-1 only	14	DNAJC25, NCKAP1, IFT80, CECR5, NUCB2, UBE2W, DAP, PTBP3, KIAA1033, RCN3, UBA3, CCDC122, ABCA5, HSPBAP1
IL-4 and IL-4+SDF-1 only	140	JARID2, ZRANB1, VRK2, ARNTL2, ANK1, AP3D1, RASSF1, ABCC9, TCF3, FBLN1, SMARCA2, CPOX, NID2, AARS, CCDC80, PSME1, SNAP23, DPYSL2, HIF1A, C20orf24, SAMHD1, E2F1, SRPX2, PLA2G15, HMOX2, LACTB, NDRG1, SLC9A3R1, NDUFC1, CPZ, TMEM109, ELK3, WNT5B, SH2B3, OAS3, ST8SIA1, AUP1, DOK1, STAT1, FHL2, PLEKHB2, RPA2, PLAGL1, KLF12, EGR1, GLT8D2, CCDC53, PTK2B, CLCC1, FAM126A, HJURP, OBSL1, EFNB2, GNAI1, MPST, CALU, SPCS3, SH3BGRL, CLUH, MORC4, TMEM106C, SLC38A2, COL4A2, NREP, OSTF1, CD36, GNS, LAMC1, ENPP2, NUMA1, NIPA2, TSPAN3, BCAS3, PTRH2, FHAD1, DEGS1, OSBPL10, SLIT2, SRD5A1, IGFBP3, SLC12A9, CHST7, SNX30, CTF1, RMND5A, KCTD15, ADAMTS1, DEPTOR, TMEM171, MGAT4B, PPAP2B, IGFBP7, ANKZF1, ATRIP, RHOBTB3, ANKRA2, C7orf60, DACT1, TAF10, PPFIBP2, PIP4K2C, SCG5, PPP2R3B, PPP1R14A, BEST1, POLR2G, RAC3, NIPA1, DCLK2, C11orf24, ATP6V0E2, PPP1R3B, PPP1R14B, LRFN4, C16orf91, RGMB, RPL15, ARHGAP1, ATG13, CLTB, TUBB6, IL17RA, ARL6IP6, RPP25, MPI, PHLDA2, SETD3, COL4A1, THSD4, TUBB4B, FAM180A, MPZL1, MFAP3L, SMG5, MIR503HG, MIF, CHMP4A, RP11-166D19.1, RPL10, RP11-572C15.6
IL-4 and_SDF-1 only	10	MGRN1, UBE3A, MTRF1, MXD4, VAMP7, TWF1, SYVN1, SMC6, MSL3P1, hsa-mir-6723
IL-4+SDF-1 and SDF-1 only	5	TARS, BTF3L4, FADS2, DEF8, FAM57A

Table 3. Selected molecular and cellular functions predicted to differ between IL-4 and SDF-1 and control, i.e. non treated hADSCs (according to Gene Ontology and GO Annotations).

**Gene Ontology ID**	**Gene Ontology Term**	**Number of All Genes in the Category**	**Number of Genes which Expression was Up- (↑) or Downregulated (↓)**	
			↑ IL-4 and SDF 1	↓ IL-4 and SDF-1
GO:0008283	cell proliferation	1186	17	10
GO:0007049	cell cycle	1441	14	12
GO:0007267	cell-cell signaling	889	11	9
GO:0048869	cellular developmental process	2513	36	27
GO:0016477	cell migration	884	16	8
GO:0030154	cell differentiation	2367	32	25
GO:0030198	extracellular matrix organization	226	9	3
